# Definition and verification of novel metastasis and recurrence related signatures of ccRCC: A multicohort study

**DOI:** 10.1002/cai2.25

**Published:** 2022-08-30

**Authors:** Aimin Jiang, Qingyang Pang, Xinxin Gan, Anbang Wang, Zhenjie Wu, Bing Liu, Peng Luo, Le Qu, Linhui Wang

**Affiliations:** ^1^ Department of Urology, Changhai Hospital Naval Medical University (Second Military Medical University) Shanghai China; ^2^ Department of Urology, Changzheng Hospital Naval Medical University (Second Military Medical University) Shanghai China; ^3^ Department of Urology, The Third Affiliated Hospital Naval Medical University (Second Military Medical University) Shanghai China; ^4^ Department of Oncology, Zhujiang Hospital Southern Medical University Guangzhou China; ^5^ Department of Urology, Affiliated Jinling Hospital Medical School of Nanjing University Nanjing China

**Keywords:** clear cell renal cell carcinoma, metastasis, recurrence, machine learning, multiple omics, single‐cell sequencing

## Abstract

**Background:**

Cancer metastasis and recurrence remain major challenges in renal carcinoma patient management. There are limited biomarkers to predict the metastatic probability of renal cancer, especially in the early‐stage subgroup. Here, our study applied robust machine‐learning algorithms to identify metastatic and recurrence‐related signatures across multiple renal cancer cohorts, which reached high accuracy in both training and testing cohorts.

**Methods:**

Clear cell renal cell carcinoma (ccRCC) patients with primary or metastatic site sequencing information from eight cohorts, including one out‐house cohort, were enrolled in this study. Three robust machine‐learning algorithms were applied to identify metastatic signatures. Then, two distinct metastatic‐related subtypes were identified and verified; matrix remodeling associated 5 (MXRA5), as a promising diagnostic and therapeutic target, was investigated in vivo and in vitro.

**Results:**

We identified five stable metastasis‐related signatures (renin, integrin subunit beta‐like 1, MXRA5, mesenchyme homeobox 2, and anoctamin 3) from multicenter cohorts. Additionally, we verified the specificity and sensibility of these signatures in external and out‐house cohorts, which displayed a satisfactory consistency. According to these metastatic signatures, patients were grouped into two distinct and heterogeneous ccRCC subtypes named metastatic cancer subtype 1 (MTCS1) and type 2 (MTCS2). MTCS2 exhibited poorer clinical outcomes and metastatic tendencies than MTCS1. In addition, MTCS2 showed higher immune cell infiltration and immune signature expression but a lower response rate to immune blockade therapy than MTCS1. The MTCS2 subgroup was more sensitive to saracatinib, sunitinib, and several molecular targeted drugs. In addition, MTCS2 displayed a higher genome mutation burden and instability. Furthermore, we constructed a prognosis model based on subtype biomarkers, which performed well in training and validation cohorts. Finally, MXRA5, as a promising biomarker, significantly suppressed malignant ability, including the cell migration and proliferation of ccRCC cell lines in vitro and in vivo.

**Conclusions:**

This study identified five robust metastatic signatures and proposed two metastatic probability clusters with stratified prognoses, multiomics landscapes, and treatment options. The current work not only provided new insight into the heterogeneity of renal cancer but also shed light on optimizing decision‐making in immunotherapy and chemotherapy.

## Graphical abstract

Multicohorts of clear cell renal cell carcinoma (ccRCC) datasets and machine learning algorithms were used to identify and verify metastatic biomarkers. Two distinctive ccRCC subgroups were uncovered and compared at multiomics level and targeting matrix remodeling associated 5 (MXRA5) could be treated as an effective approach to suppress ccRCC progression.

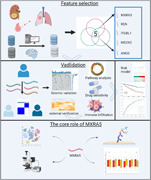

**Abbreviations**
ANO3: anoctamin 3
APM: antigen processing machinery
CCK‐8: Cell Counting Kit‐8
CCLE: Cancer Cell Line Encyclopedia
ccRCC: clear cell renal cell carcinoma
CNVs: copy number variations
CPTAC: Clinical Proteomic Tumor Analysis Consortium
DEGs: differentially expressed genes
EMT: epithelial–mesenchymal transition
GEO: Gene Expression Omnibus
GO: Gene Ontology
GSEA: gene set enrichment analysis
GSVA: gene set variation analysis
IC_50_
: half‐maximal inhibitory concentration
ITGBL1: integrin subunit beta‐like 1
KEGG: Kyoto Encyclopedia of Genes and Genomes
LASSO: least absolute shrinkage and selection operator
MEOX2: mesenchyme homeobox 2
MTCS1: metastatic cancer subtype 1
MTCS2: metastatic cancer subtype 2
MXRA5: matrix remodeling associated 5
NSCLC: nonsmall cell lung cancer
OS: overall survival
PAC: proportion of ambiguous clustering
PFS: progression‐free survival
RC: renal cancer
REN: renin
RF: random forest
RT‐qPCR: reverse transcription‐quantitative polymerase chain reaction
SVM: support vector machine
TCGA: The Cancer Genome Atlas
TIDE: tumor immune dysfunction and exclusion
TPM: transcripts per kilobase million

## 1 INTRODUCTION

Renal cancer (RC) is one of the most fatal cancers in the urogenital system, with an incidence only secondary to that of prostate and bladder cancer [1]. According to the Latest Global Cancer Statistics reports, there are nearly 431,288 new RC cases and 179,368 RC‐related deaths per year [2]. Clear cell renal cell carcinoma (ccRCC) represents the most common subtype of RC, accounting for nearly 80% of cases [3]. Although targeted therapy and immunotherapy have significantly improved the prognosis of ccRCC, nearly 1/3 of patients present with metastasis at first diagnosis, and approximately half of early‐stage cases develop distant metastasis even after partial or radical nephrectomy [4]. Once patients develop metastasis, the 5‐year survival rate decreases to lower than 10% [5]. The common metastatic sites are the bone and lung, and the number of metastases is highly variable and determines prognosis and therapy response [1, 6]. Therefore, it is essential to decipher the mechanism of metastasis and identify specific biomarkers for ccRCC patients.

Recently, accumulated studies have indicated that genetic and epigenetic alterations are closely related to the prognosis and treatment of ccRCC [7, 8, 9, 10]. With the progress in high‐throughput sequencing and bioinformatics, several prognostic models have been developed [11, 12, 13, 14]. In addition, there are numerous signatures based on microRNA, messenger RNA, long noncoding RNA, and methylation sites as candidate biomarkers in ccRCC [15, 16, 17]. Unfortunately, because of inappropriate machine‐learning algorithms, underutilized cohort information, and absent validation cohorts, most of these signatures fail to apply to clinical practice. In addition, almost all models are designed to predict survival outcomes, and most metastatic‐related signatures are filtered via survival time, which is not applicable to evaluating the metastatic probability. In clinical work, positron emission tomography–computed tomography is an approach used to detect the metastatic sites of ccRCC patients, but it is not a routine screen for early‐stage populations and has a misdiagnosis rate [18]. Nevertheless, the results are determined by abdominal imaging and clinicopathology, which may lead to an approximate margin of error of 20% [19, 20]. To address these limitations, the development of a specific model to evaluate the metastatic tendency among ccRCC patients, especially in the early stage, is urgently needed.

In this study, we attempted to apply machine‐learning algorithms to decipher ccRCC‐related metastatic biomarkers and construct an early risk stratification classifier of ccRCC, which might help clinical practitioners assess recurrence probability, metastasis, survival time, and drug sensitivity in ccRCC. Our work may facilitate optimized management and further improve the clinical outcomes of ccRCC patients.

## 2 MATERIALS AND METHODS

### 2.1 Data collection and processing

Most ccRCC datasets in our study were retrospectively enrolled in public databases, including Gene Expression Omnibus (GEO; GSE105288: *n* = 35, primary RC tissue = 9, metastatic RC tissues = 26; GSE22542: *n* = 68, primary cancer tissues = 27, metastatic RC tissues = 41; GSE23629: *n* = 32, primary cancer tissues = 16, metastatic RC tissues = 16; GSE85258: *n* = 30, primary cancer tissues = 15, metastatic RC tissues = 15); The Cancer Genome Atlas‐kidney renal clear cell carcinoma (TCGA‐KIRC); Cancer Cell Line Encyclopedia (CCLE); and single‐cell sequencing data from previous research [21, 22, 23, 24, 25]. The Changhai cohort containing primary and metastasis RC sites (including RNA sequence and proteomics datasets of 13 primary and 14 metastatic tissues) was also applied for validation. For cohorts from public databases, institutional review board approval and informed consent were not required. Level‐3 transcriptome and clinical information were downloaded from the TCGA [26]. RNA‐sequencing data of count and transcripts per kilobase million (TPM) from the TCGA‐KIRC cohort were obtained from the Genomic Data Commons (GDC) database (https://portal.gdc.cancer.gov/); the former was used for differential expression analysis and further transformed to log2 (TPM + 1) for subsequent analysis. All expression data were normalized before analysis. Patients were excluded if they 1) did not have prognostic information and 2) died within 30 days.

### 2.2 Batch removal and metastasis‐related signature identification

The R package “sva” was used to remove the batch effect from different datasets, and samples with primary or metastatic annotation were maintained for further analysis. First, differentially expressed genes (DEGs) between primary and metastatic sites were detected with the threshold *p* < 0.05 and |log fold‐change|>1.2). The R package “carcet” was used to divide the combined dataset into training data and testing data (ratio = 7:3). Then, three machine‐learning algorithms, including least absolute shrinkage and selection operator (LASSO) logistic, support vector machine (SVM), and random forest (RF), were introduced to identify metastatic relevant signatures of ccRCC.

### 2.3 Identification of the optimal cluster number

After obtaining five metastatic signatures, we aimed to apply them to decipher the heterogeneity and metastasis tendency of ccRCC. This analysis was performed via the R package “ConsensusClusterPlus” to identify optimal cluster number k (detailed parameters: reps = 100, pItem = 0.8, clusterAlg = “km,” distance = “euclidean”). Subsequently, we applied the consensus score matrix, cumulative distribution function curve, the proportion of ambiguous clustering (PAC) score, and Nbclust to determine the optimal cluster number. Finally, ccRCC patients were grouped into two distinct subtypes named metastatic cancer subtype 1 (MTCS1) and type 2 (MTCS2), and *k* = 2 was treated as the optimal cluster number.

### 2.4 Enrichment analysis between subgroups

The R package “DEseq. 2” was used to identify DEGs between MTCS1 and MTCS2, and the thresholds were set to adjusted *p* < 0.01 and abstract log fold‐change >2 [27]. After calculating DEGs, the R package “ClusterProfiler” was used to perform Gene Ontology (GO), Kyoto Encyclopedia of Genes and Genomes (KEGG) pathway, gene set enrichment analysis (GSEA), and gene set variation analysis (GSVA) to explain differences in the biological function and molecular mechanism between MTCS1 and MTCS2 [28]. All gmt files used for enrichment analysis were downloaded from Molecular Signatures Database (MSigDB) (https://www.gsea-msigdb.org/gsea/index.jsp) and ConsensusPathDB (http://cpdb.molgen.mpg.de/) databases [29, 30].

### 2.5 Differences in immune infiltration and therapy response

We used multiple immune cell infiltration algorithms, including CIBERSORT, EPIC, MCPCOUNTER, QUANTISEQ, TIMER, and XCELL, to calculate immune cell enrichment and cellular component scores in ccRCC tissues and compare differences in immune components between subgroups [31, 32, 33, 34, 35]. In addition, single‐sample GSVA was introduced to validate these differences between MTCS1 and MTCS2 [36]. The R package “ESTIMATE” was used to evaluate the stromal and immune scores. The tumor immune dysfunction and exclusion (TIDE, http://tide.dfci.harvard.edu/) algorithm was applied to illustrate the different immunotherapy sensitivities [37].

### 2.6 Mutation spectrum between subtypes

Somatic datasets of ccRCC were analyzed and visualized via the R package “Maftools” [38]. Using correlation functions, the tumor mutation panorama, base transitions and transversions, single nucleotide variants, mutation rates of alleles, copy number mutations, and mutually exclusive or coexisting mutations were calculated. Drug and gene interactions and the differences in oncogenic signaling pathways between different subsets were also analyzed through transformation analysis. We also analyzed recurrent extensive and focal somatic copy number alterations via the GISTIC 2.0 (https://cloud.genepattern.org/gp/pages/index.jsf) algorithm [39, 40].

### 2.7 Drug susceptibility prediction

The R package “pRRophetic” was used to estimate the half‐maximal inhibitory concentration (IC_50_) and cross‐validate the predicted results. In addition, CellMiner (https://discover.nci.nih.gov/cellminer/home.do), CCLE (https://sites.broadinstitute.org/ccle), and Genomics of Drug Sensitivity in Cancer (GDSC) (https://www.cancerrxgene.org/) databases were introduced to compare the different sensitivities between ccRCC cell lines [41, 42, 43]. Spearman's correlation coefficient was used to identify the association between gene expression and drug sensitivity. A positive correlation indicates that high expression of the signature might lead to resistance, and low expression may lead to sensitivity to targeted therapy.

### 2.8 Construction of a risk prediction model related to copper‐induced cell death

First, we used subgroup‐related biomarkers and overall prognostic information from the TCGA‐KIRC cohort to perform a univariate Cox regression analysis and select survival‐related signatures. Then, the random survival forest variable hunting algorithm was applied to identify crucial signatures. Finally, a risk scoring model was constructed using the best combination of prognostic signatures. The JAPAN‐ccRCC cohort was used to validate the risk model, and patients in the training and test cohorts were divided into high‐ and low‐risk subgroups according to median risk scores.

### 2.9 Validation of differential matrix remodeling associated 5 expression

We used reverse transcription‐quantitative polymerase chain reaction (RT‐qPCR) and immunohistochemical staining to validate differential matrix remodeling associated 5 (MXRA5) expression between paired tumor and normal tissues (including 40 paired tissues from Changhai Hospital). Primer sequences for RT‐qPCR were as follows: MXRA5 (forward primer: CCTTGTGCCTGCTACGTCC, reverse primer: TTGGTCAGTCCTGCAAATGAG) and glyceraldehyde 3‐phosphate dehydrogenase (forward primer: GGAGCGAGATCCCTCCAAAAT, reverse primer: GGCTGTTGTCATACTTCTCATGG). An MXRA5 antibody (cat number: PA5‐37267) was purchased from Thermo Fisher Group Ltd. The detailed procedures were described in our previous studies [13, 44, 45].

### 2.10 Investigation of MXRA5 biological function

All cell lines used in our study were purchased from the American Type Culture Collection. Cell lines were cultured according to standard protocols. The plasmid for the MXRA5 knockdown was chemically synthesized by Shanghai GeneChem Co., Ltd. 786‐0 and ACHN cells were cultured in six‐well culture dishes at a 60% density and then infected with lentivirus. All transfections were performed with 4 µg/mL Polybrene (H8761; Solarbio Inc.) and lasted for 12 h. The screening was conducted with 2 µg/mL puromycin (P8833; Sigma Inc.) for 3 days to acquire stably transfected cells. RT‐qPCR and western blotting were applied to verify the knockdown efficiency. Cell Counting Kit‐8 (CCK‐8) was used to detect cell viability between negative control and MXRA5 knockdown groups. The scratch assay and transwell migration assay were used to evaluate cell migration ability. A colony‐forming experiment was conducted to determine the ability of reproduction. A nude mouse xenograft tumor model assay was applied to investigate the effect of MXRA5 on tumor proliferation. The detailed procedure of these experiments was described in our previous study. All experiments mentioned above were carried out in three replications and repeated three times.

### 2.11 Statistical analysis

All data processing, statistical analysis and plotting were performed via R software (version 4.0.4). Kruskal–Wallis and Wilcoxon test were used to compare continuous variables. Chi‐square test was applied to compare categorical variable. R package “survival” was used to perform Cox regression and Kaplan–Meier analysis. The receiver operating characteristic curve (ROC) applied to predict binary categorical variables was implemented via R package “pROC”. The time‐dependent area under the ROC curve (AUC) for survival variables was conducted by the package “timeROC”. All statistical tests were two‐sided, and *p* < 0.05 was considered statistically significant.

## 3 RESULTS

### 3.1 Batch effects removal and data preparation

**Figure 1:**
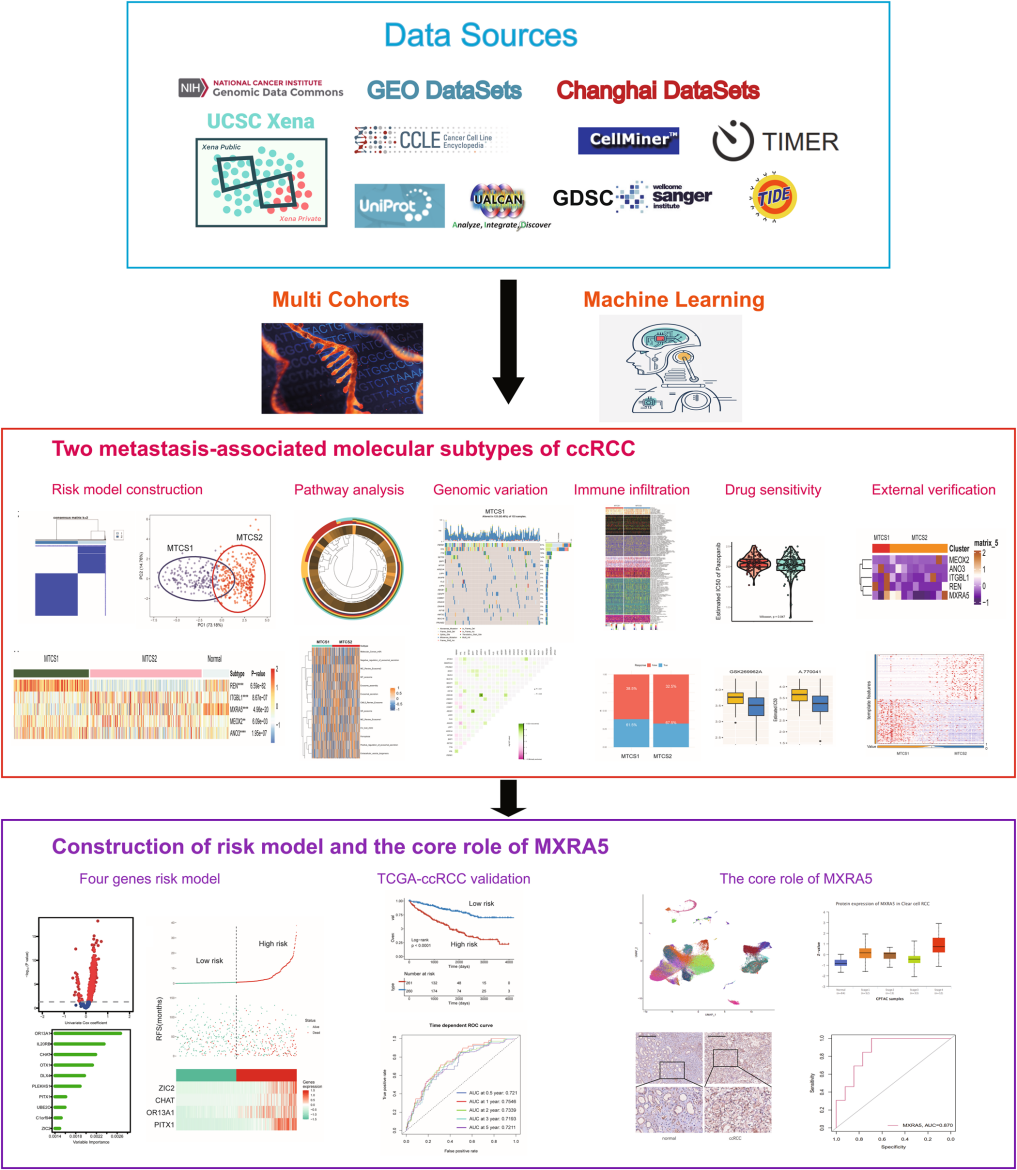
Overall workflow diagram of the study. ANO3, anoctamin 3; AUC, area under the curve; ccRCC, clear cell renal cell carcinoma; GEO, Gene Expression Omnibus; ITGBL1, integrin subunit beta‐like 1; MEOX2, mesenchyme homeobox 2; MTCS1, metastatic cancer subtype 1; MTCS2, metastatic cancer subtype 2; MXRA5, matrix remodeling associated 5; REN, renin; ROC, receiver operating characteristic; TCGA, The Cancer Genome Atlas.

**Figure 2:**
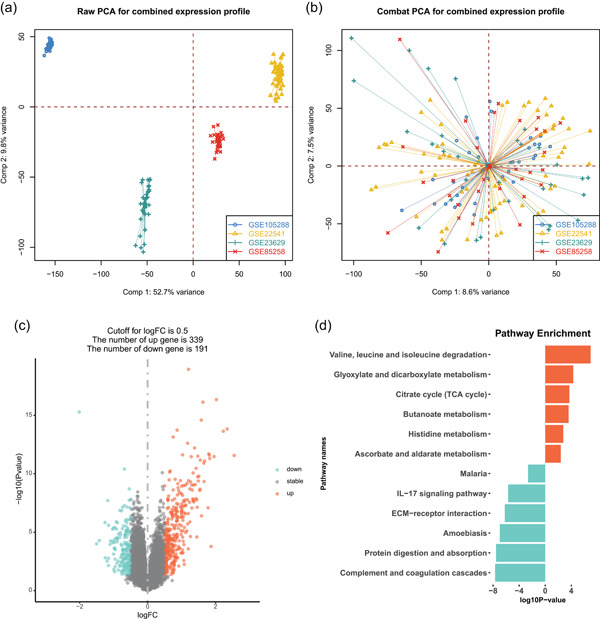
Differentially expressed signatures between primary and metastatic sites of ccRCC. (a, b) PCA plot before and after batch effect removal. (c) Volcano plot of differentially expressed signatures. (d) KEGG analysis of differentially expressed signatures. ccRCC, clear cell renal cell carcinoma; ECM, extracellular matrix; FC, fold change; IL‐17, interleukin‐17; KEGG, Kyoto Encyclopedia of Genes and Genomes; PCA, principal component analysis; TCA, tricarboxylic acid.

The workflow of this study was depicted in Figure 1. Four microarray datasets (GSE105288, GSE22541, GSE23629, and GSE85258) were downloaded from the GEO database. After quality control, the “combat” function of the “sva” package was used to remove the batch effect of nonbiological technical biases among different datasets. The batch effect was removed to some extent, as depicted in Figure 2a,b. We then analyzed DEGs between metastatic and primary RC tissues, resulting in the identification of 530 DEGs (Figure 2c). The functions of DEGs were mainly enriched in valine, leucine, and isoleucine degradation, glyoxylate and dicarboxylate metabolism, citrate cycle, complement and coagulation cascade, and protein digestion and absorption (Figure 2d).

### 3.2 Metastatic signature identification

**Figure 3:**
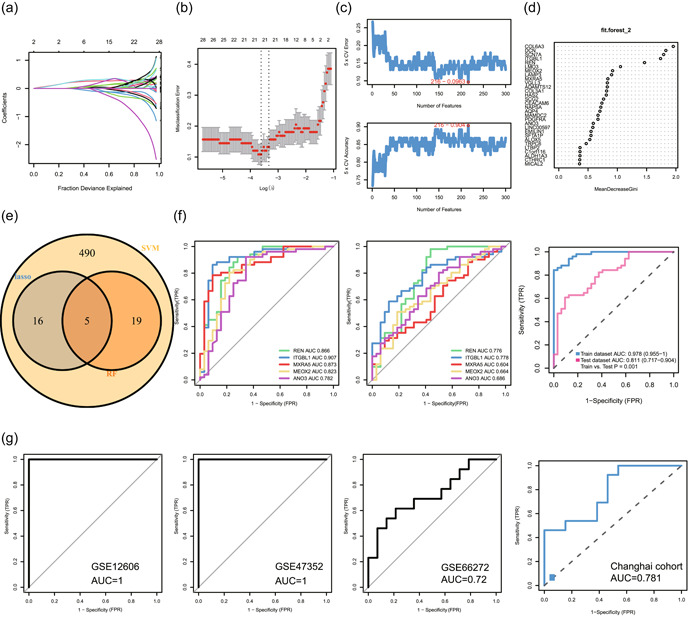
Three algorithms are used for feature selection. (a, b) LASSO, (c) SVM–RFE, and (d) RF algorithms in the discovery cohort. (e) Intersections of features from the three algorithms. (f) Receiver operating characteristic (ROC) curves for the ability of the five signatures and a combination of the five signatures in training and testing cohorts. (g) ROC curve for the combination of the five signatures in GSE12606, GSE47352, GSE66272, and Changhai cohorts. ANO3, anoctamin 3; AUC, area under the curve; FPR, false positive rate; ITGBL1, integrin subunit beta‐like 1; LASSO, least absolute shrinkage and selection operator; MEOX2, mesenchyme homeobox 2; MXRA5, matrix remodeling associated 5; REN, renin;  RF, random forest; RFE, recursive feature elimination; SVM, support vector machine; TPR, true positive rate.

In total, 530 DEGs were introduced to select signatures for classifying nonmetastatic and metastatic tissues. LASSO logistic, SVM–recursive feature elimination, and RF algorithms were applied to identify metastatic‐related genes (Figure 3a–d). After obtaining metastatic signatures selected using the three algorithms, five signatures (renin [REN], integrin subunit beta‐like 1 [ITGBL1], MXRA5, mesenchyme homeobox 2 [MEOX2], and anoctamin 3 [ANO3]) were finally identified (Figure 3e). Then, each gene was introduced to test the discrimination ability of metastasis in training and testing cohorts, and REN showed the most robust assessment ability in the two cohorts (Figure 3f). In addition, five signatures were combined to evaluate the prediction ability, and the area under the curve (AUC) of the combined signature reached 0.978 in the training cohort and 0.811 in the testing cohort (Figure 3f). Furthermore, to evaluate the robustness of this model, four out‐house cohorts, including a cohort from Changhai Hospital, were enrolled. The AUC was 1 in GSE47353 and GSE12606, 0.72 in GSE66272, and 0.781 in the Changhai cohort. All results suggested the specificity and reproductivity of our model (Figure 3g).

### 3.3 Regulation mechanism of metastatic signatures

Because the five metastatic signatures displayed a significant role in ccRCC progression, we then systematically analyzed the regulation of these signatures at the DNA methylation and RNA modification level. As illustrated in Supporting Information: Figure S1A, the expression levels of ITGBL1 and MEOX2 were negatively correlated with the methylation degree. REN and ANOX3 expression levels were significantly related to RNA modification (Supporting Information: Figure S1B). Together, these results indicated that epigenetic modifications might regulate metastatic events in ccRCC.

### 3.4 Identifying two distinct metastatic subtypes

**Figure 4:**
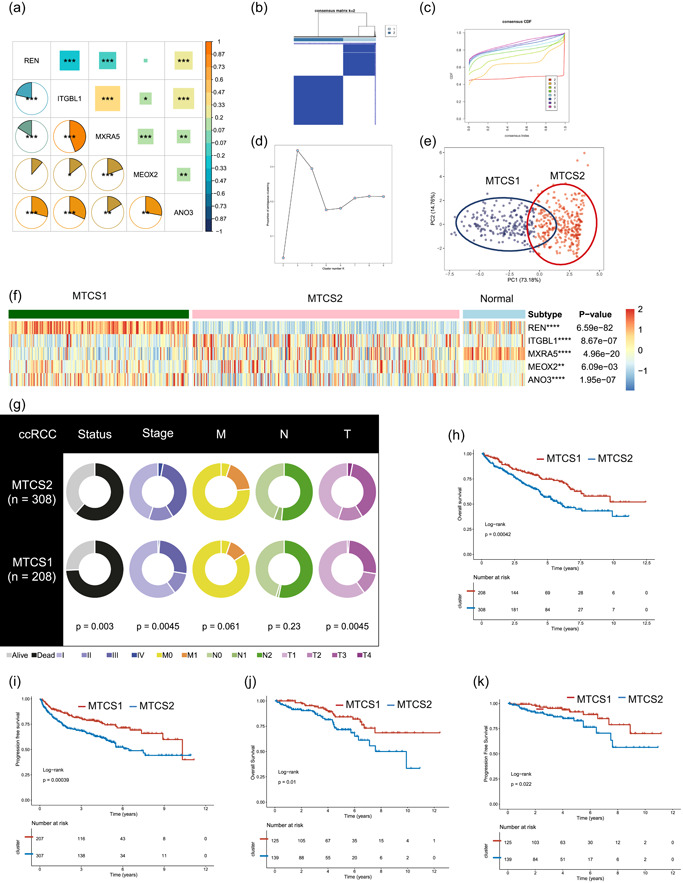
Establishment of two clusters of metastasis‐related signatures in ccRCC. (a) Correlation of the five metastasis signatures via Pearson and Spearman algorithms. (b) Consensus cluster matrix of ccRCC tumor samples when *k* = 2. (c) The cumulative distribution function curves also indicated *k* = 2 as the optimal cluster number. (d) The proportion of ambiguous clustering scores. The optimal *k* number was 2. (e) Two‐dimensional principal component plot by the matrix containing the five metastasis‐related signatures from the ccRCC cohort. The blue dots represent MTCS1, and the red dots represent MTCS2. (f) The expression heatmap of the five metastasis‐related signatures among MTCS1, MTCS2, and normal tissues. (g) Difference of clinical features between MTCS1 and MTCS2. (h, i) Survival analysis for overall survival and progression‐free survival of the two subtypes in the TCGA–ccRCC dataset. (j, k) Survival analysis for overall survival and progression‐free survival of the two subtypes in the stage T1 group. ANO3, anoctamin 3; ccRCC, clear cell renal cell carcinoma; ITGBL1, integrin subunit beta‐like 1; MEOX2, mesenchyme homeobox 2; MTCS1, metastatic cancer subtype 1; MTCS2, metastatic cancer subtype 2; MXRA5, matrix remodeling associated 5; REN, renin; TCGA, The Cancer Genome Atlas.

Since the five metastatic signatures might play a pivotal role in the progression of ccRCC, we investigated their impact on ccRCC's heterogeneity. We first calculated the expression relationship of the five signatures and found that the expression level of ITGBL1 was significantly positively correlated with that of MXRA5 (Figure 4a). The TCGA–ccRCC samples were classified into two subtypes using an unsupervised clustering method based on a metastatic signature matrix. The optimal classification number was validated, and the PAC method was used to test the robustness of the clustering result. Consequently, the two subtypes were named MTCS1 and MTCS2 (Figure 4b–e). Moreover, we found that metastatic signatures displayed a significantly different expression pattern between subtypes and normal tissues (Figure 4f). Compared with the MTCS1 subtype, the MTCS2 subtype exhibited a higher T stage and shorter OS and PFS times (Figure 4g–i), especially in the T1 stage subgroup (Figure 4j,k).

### 3.5 Pseudotiming analysis

**Figure 5:**
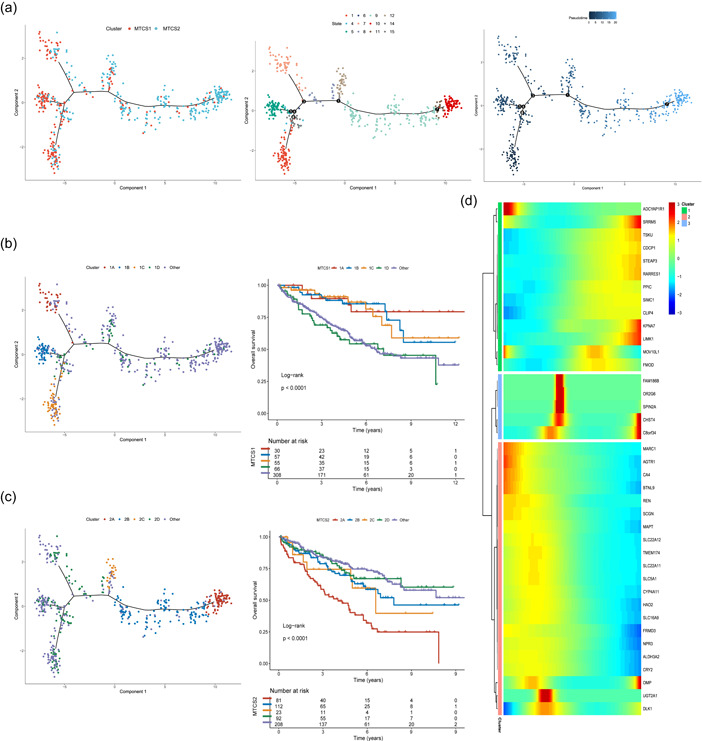
The progression landscape between MTCS1 and MTCS2. (a) The distribution landscape of MTCS1 and MTCS2: Each point represents a patient with colors corresponding to the subtype defined previously (left); subtypes of ccRCC clustered by state (middle); pseudotime trajectories from MTCS1 to MTCS2 (right). (b) Patients of the MTCS1 subtype could be further stratified into four subgroups with distinct prognoses. (c) Patients of the MTCS2 subtype could be further stratified into four subgroups with distinct prognoses. (d) Heatmap showing the top signatures that affected the progression from MTCS1 to MTCS2. MTCS1, metastatic cancer subtype 1; MTCS2, metastatic cancer subtype 2.

The expression profiles in MTCS1 and MTCS2 were selected to construct the progression landscape of ccRCC. Consistent with the defined subtypes, most patients could be segregated into two distinct clusters and different states (Figure 5a). The location of individual patients in the trajectory plot represented the metastatic characteristics. For instance, MTCS1 and MTCS2 were distributed at the opposite ends of the horizontal axis (Figure 5a). Therefore, we hypothesized that the horizontal axis in the trajectory plot represented the progression of metastasis. However, the vertical coordinate of the developmental trajectory plot appeared to be more complex and might reflect several characteristics. The trajectory plot further revealed intracluster heterogeneity within each subtype. For instance, MTCS1 could be further divided into four subgroups based on their location in the trajectory plot with distinct prognoses (Figure 5b). Similar results were obtained in MTCS2. The results of prognosis analysis showed four distinct subtypes in the risk landscape within MTCS2 because 2A showed a poorer prognosis (Figure 5c). These results indicate that clustering analysis provides a complementary value to previously identified ccRCC subtypes. Because MTCS1 and MTCS2 maintained an evolutionary relationship, we performed pseudotime analysis and identified the key meditators involved in the transformation from MTCS1 to MTCS2 (Figure 5d).

### 3.6 DEGs and functional analysis

**Figure 6:**
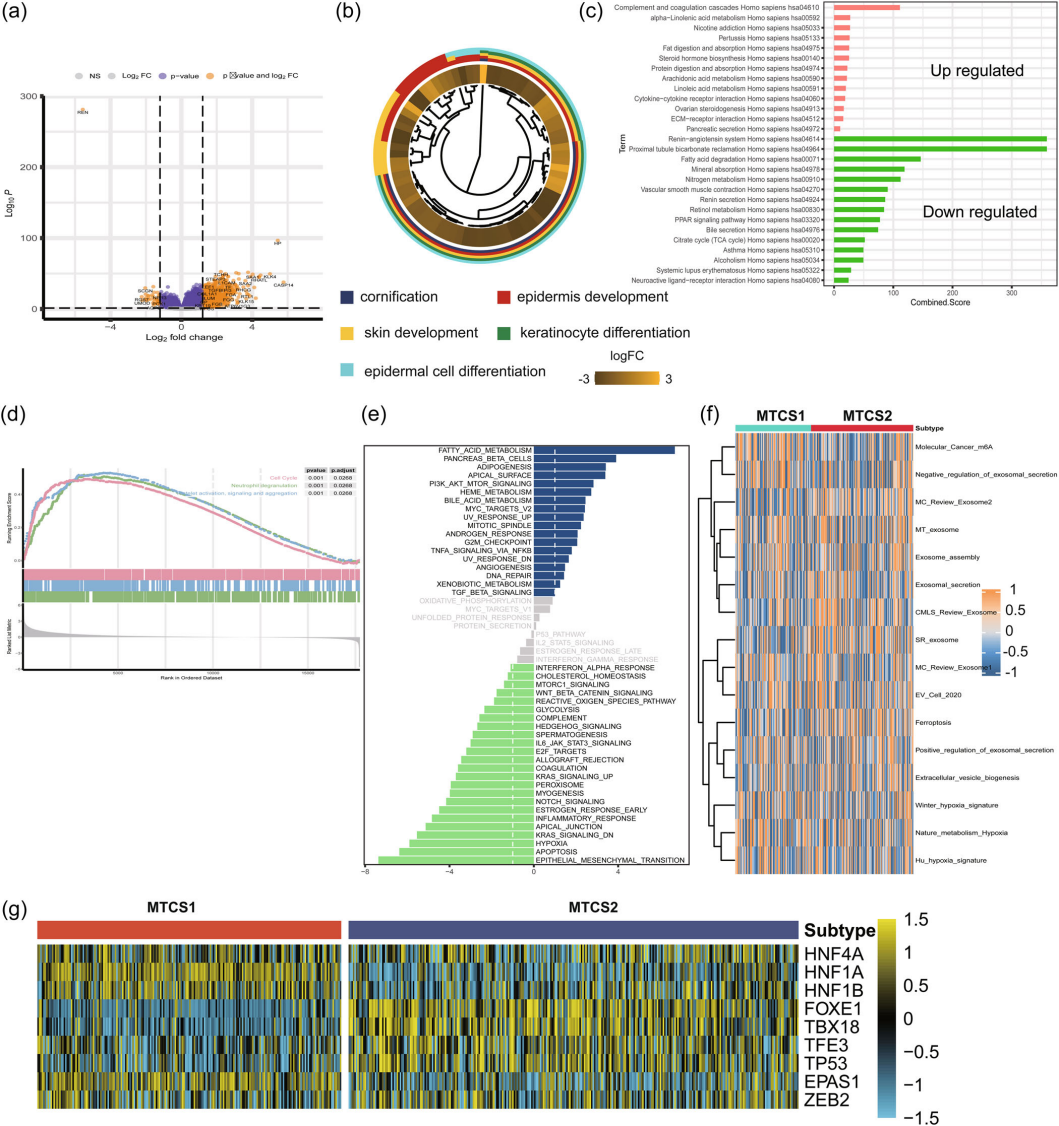
Functional enrichment analysis of ccRCC subtypes. (a) Volcano plot of differentially expressed genes between subgroups. (b) GO enrichment analysis, (c) KEGG, (d) GSEA, (e) GSVA, and (f) ssGSEA analysis between the two subtypes. (g) Heatmap of transcription factor regulation between subtypes. Yellow represented transcription factor activation. Blue represented transcription factor inhibition. ccRCC, clear cell renal cell carcinoma; GO, gene ontology; GSEA, gene set enrichment analysis; GSVA, gene set variation analysis; KEGG, Kyoto Encyclopedia of Genes and Genomes; MTCS1, metastatic cancer subtype 1; MTCS2, metastatic cancer subtype 2; ssGSEA, single‐sample GSEA.

The expression profiles of ccRCC were analyzed to identify metastatic‐related DEGs, which included 39 downregulated and 926 upregulated genes (Figure 6a). Combined with previous clinical outcomes and metastatic tendencies, we next investigated the difference in biological functions and hallmarks of DEGs. GO analysis demonstrated that DEGs were mainly enriched in cornification, epidermis development, skin development, keratinocyte differentiation, and epidermal cell differentiation for biological processes; collagen‐containing extracellular matrix, cornified envelope, blood microparticle, intermediate filament and intermediate filament cytoskeleton for cellular components; and extracellular matrix structural constituent, serin‐type endopeptidase inhibitor activity, peptidase inhibitor activity, receptor–ligand activity, and signaling receptor activator activity for molecular functions (Figure 6b, Supporting Information: Figure S2A). KEGG results demonstrated that complements and coagulation cascades, alpha−linolenic acid metabolism, nicotine addiction, and pertussis were activated in MTCS2, whereas REN−angiotensin system, proximal tubule bicarbonate reclamation, fatty acid degradation, and nitrogen metabolism were inhibited (Figure 6c). GSEA results indicated that DEGs were mainly involved in cell cycle, neutrophil degranulation, platelet activation, signaling, and aggregation pathways (Figure 6d). Compared with MTCS1, MTCS2 showed activated fatty acid metabolism, adipogenesis, apical surface, and phosphatidylinositol‐3‐kinase–protein kinase B (Akt)–mammalian target of rapamycin signaling but inhibited epithelial–mesenchymal transition (EMT), apoptosis, KRAS pathway, and inflammatory responses (Figure 6e). Furthermore, we applied GSVA algorithms to investigate other signatures, which displayed that MTCS1 and MTCS2 owned different activation degrees of molecular cancer m6A, negative regulation of exosome secretion, microvesicle‐induced exosome ferroptosis, and metabolism signatures (Figure 6f). Together, these results indicate that renal cell metastasis involves multiple biological components, from genomic and epigenetic factors to metabolism. The R package “RTN” was used to assess transcription factor activity between subgroups. As shown in Figure 6g, MTCS1 displayed higher HNF1A, HNF1B, and EPAS1 activity, whereas MTCS2 exhibited higher FOX1, TBX18, TFE2, and TP53 activity (Figure 6g). Metabolic processes play crucial roles in ccRCC. We observed the activation of several metabolic pathways in MTCS2, including cyclooxygenase arachidonic acid metabolism, prostanoid biosynthesis, glycosphingolipid biosynthesis, and thromboxane biosynthesis. In MTCS1, glycerolipid metabolism, histidine metabolism, and fatty acid degradation were stimulated (Supporting Information: Figure S2B). All results indicated that metabolic reprogramming might shape the ccRCC metastasis environment.

### 3.7 Comparison of the immune landscapes between subgroups

**Figure 7:**
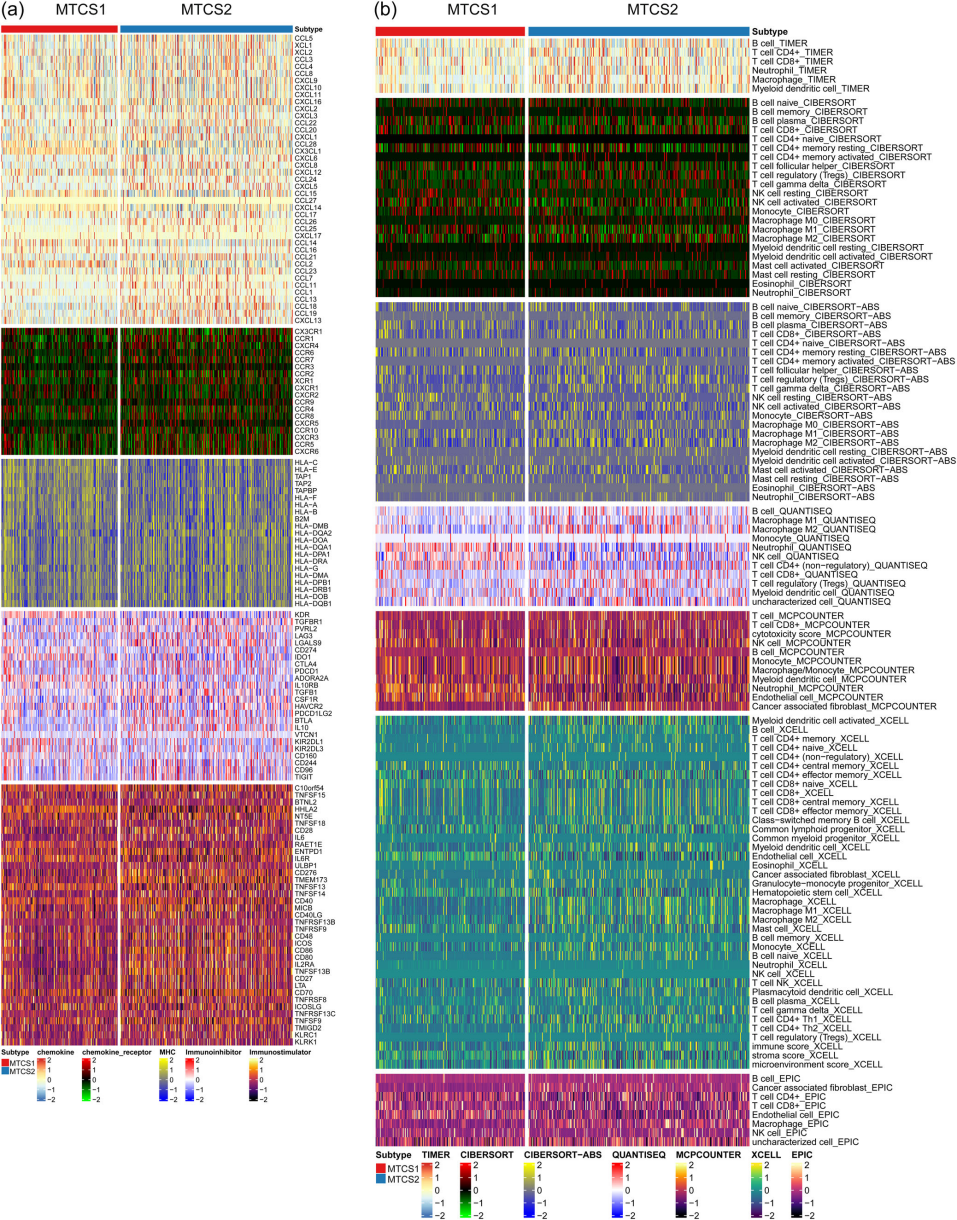
Identification of immune landscapes. (a) Heatmap of immune signatures, including chemokine, chemokine receptor, MHC, immunoinhibitory and immunostimulatory, between subgroups. (b) Heatmap of tumor‐related infiltrating immune cells based on TIMER, CIBERSORT, CIBERSORT‐ABS, QUANTISEQ, MCPCOUNTER, XCELL, and EPIC algorithms between subgroups. MHC, major histocompatibility complex; MTCS1, metastatic cancer subtype 1; MTCS2, metastatic cancer subtype 2.

**Figure 8:**
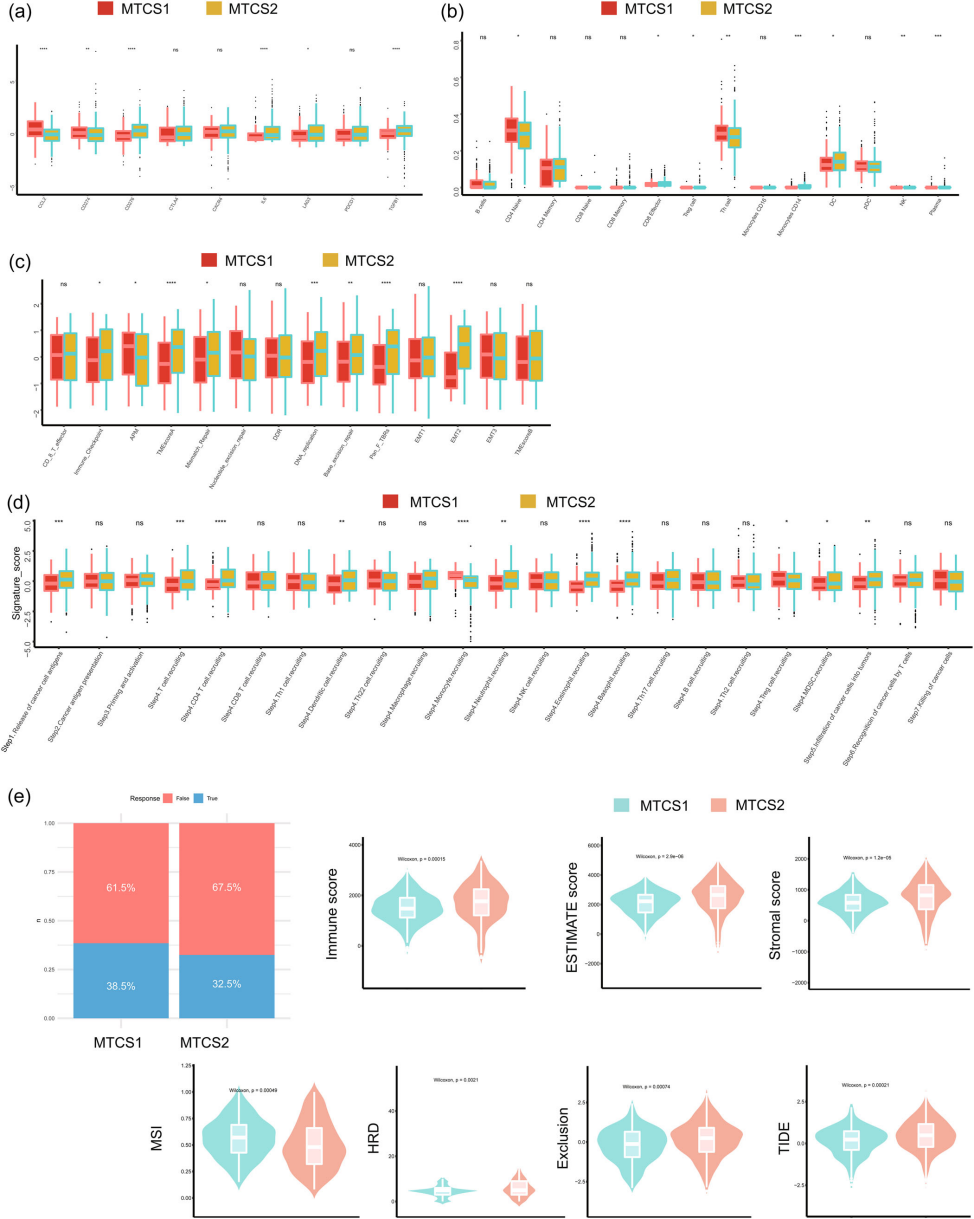
Landscapes of specific immune components and immune function scores. (a) Different expression levels of the immune checkpoint inhibitors, (b) normalized enrichment scores of immune cells, (c) immune signatures, and (d) anticancer immune response between subgroups. (e) Differences in response to immune checkpoint inhibitor treatment based on the TIDE algorithm and immune components based on the estimation algorithm. HRD, homologous recombination deficiency; MSI, microsatellite instability; MTCS1, metastatic cancer subtype 1; MTCS2, metastatic cancer subtype 2; ns, not significant; TIDE, tumor immune dysfunction and exclusion.

To investigate the relationship between metastasis and tumor immunity, we first calculated the correlation between metastatic signatures and immune cell infiltration degree. Supporting Information: Figure S3 showed that REN was almost negatively correlated with all immune cells, and ITGBL1 was positively correlated with most immune cells, which indicated that these signatures might perform distinct roles in the immune microenvironment. Figure 7a showed that chemokine, chemokine receptor, major histocompatibility complex, immunoinhibitory, and immunostimulatory signatures were differentially expressed between subgroups. Furthermore, using several immune algorithms, we compared the immune landscape between MTCS1 and MTCS2 (Figure 7b). Interestingly, MTCS1 displayed a higher infiltration of M1 macrophages, while MTCS2 contained a higher infiltration of M2 macrophages. Neutrophil infiltration was higher in MTCS1 compared with that in MTCS2 for almost all algorithms. The expression level of nine immune checkpoint inhibitor genes was compared between MTCS1 and MTCS2 (Figure 8a). Most of these genes (CD276, IL6, LAG3, and TGFB1) were upregulated, whereas CCL2 and CD274 were downregulated in MTCS2. Through TIP databases, we found that CD4 naïve and helper T cell infiltration were higher in MTCS1 subgroups, whereas CD8 effector T cell, regulatory T cell, monocyte, CD14 T cell, dendritic cell, natural killer cell, and plasma cell infiltration were higher in MTCS2 (Figure 8b). Additionally, we found higher immune checkpoint levels, tumor microenvironment (TME) scores, mismatch repair, DNA replication, transforming growth factor (TGF)‐β response, and EMT scores but lower antigen processing machinery (APM) scores in MTCS2 (Figure 8c). We also evaluated the tumor immunophenotype because the anticancer immune response can be conceptualized as a series of stepwise events. We found that MTCS2 displayed higher activity in setp1_Release of cancer antigens, step4_T cell recruiting, step4_Dentritic cell recruiting, step4_Neutrophil recruiting, step4_Eosinophil recruiting, step4_Basophil recruiting, step4_MDSC recruiting, and step5_inflitration of cancer cells into tumors but lower activity in step4_Monocyte recruiting and step4_Treg cell recruiting (Figure 8d). Furthermore, a lower immune checkpoint blockade therapy response rate was detected in MTCS2 subgroups. Moreover, all estimate scores (including stromal, immune, and estimated scores), homologous recombination deficiency, immune exclusion, and TIDE scores were significantly higher, and the microsatellite instability score was lower in MTCS2 (Figure 8e).

### 3.8 Comprehensive and integrated genomic characteristics between subgroups

Because transcriptional alterations between MTCS1 and MTCS2 were investigated, we then aimed to describe the disparity at the genomic level. The mutation rate was higher in MTCS1 (123/133, 92.48% in MTCS1 vs. 153/188, 81.38% in MTCS2), and the top 20 most frequently mutated genes were illustrated in Supporting Information: Figure S4A. Among them, the mutation rates of PBRM1, VHL, TTN, SETD2, and BAP1 were different between MTCS1 and MTCS2 (Supporting Information: Figure S4B). We also found that most mutated signatures could be treated as protective factors in MTCS2 (Supporting Information: Figure S4C), whereas tumor mutation burden showed no significant difference between subgroups (Supporting Information: Figure S4D). Next, we investigated the co‐occurring and exclusive mutations of the top 25 frequently mutated genes via the “CoMEt” algorithm and found that MTCS1 and MTCS2 contained different comutation landscapes (Supporting Information: Figure S4E,F). These included coexisting BAP1‐ABCB1 and ARID1A‐DNAH9 mutation models in MTCS1 and PBRM1‐USH2A, PBRM1‐SETD2, BAP1‐MUC16, HMCN1‐ADGRV1, CSMD3‐ERBB4, CSMD3‐NEB, CSMD3‐XIRP2, PYR1‐USH2A, and XIRP2‐ERBB4 mutation models in MTCS2, suggesting that they probably played a redundant role in the same pathway and harbored selective advantages between them to maintain more than one copy of the mutations (Supporting Information: Figure S4E,F). Among the five metastatic signatures mentioned above, REN was only mutated in MTCS2 compared with the MTCS1 subgroup (Supporting Information: Figure S4G).

In addition to the mutation pattern, we investigated differences in the copy number variations (CNVs) between subtypes. The GISTIC2.0 algorithm was used to determine the amplification and deletion of copy numbers on chromosomes. Compared with MTCS1, MTCS2 had a higher level of copy number alterations (Supporting Information: Figure S5A). The detailed results showed that both MTCS1 and MTCS2 had frequent CNVs in the regions of Chr3 and Chr5, suggesting that CNVs may play a significant role in the progression of ccRCC. The recurrent CNVs in MTCS2 included the amplification of 5q35.3 (CANX, CLTB, and DBN1), 5q23.2 (CSNK1G3, PPIC, and SNX2), and 4q32.1 (ETFDH, PPID, and RAPGEF2) and the deletion of 9q23 (PTPRD), 9p21.3 (CDKN2B and CDKN2A), and 2q37.3 (AGCT, KIF1A, and BOK). The CNVs in MTCS1 were mainly associated with tumor cell proliferation, such as the amplification of 5q35.1 (STC2), 5q15 (EII2), and 5q21.3(RAB9BP1) and the deletion of 3p25.3 (VGLL4 and ATG7), 2q35 (MREG), and 2q37.1 (AGXT, ALPI, and ALPP) (Supporting Information: Figure S5B‐D). The above results suggest that MTCS1 and MTCS2 harbor distinct CNV events, which may lead to different immune infiltration and metastatic probability.

### 3.9 Drug sensitivity between the two subgroups

The GDSC database was applied to evaluate the chemotherapy response of the two subtypes to commonly used drugs, including saracatinib and sunitinib. As shown in Supporting Information: Figure S6A, MTCS2 subgroups were more sensitive to these drugs than MTCS1. Moreover, the sensitivity to several potential prodrugs with clinical application potential was compared between MTCS1 and MTCS2. As illustrated in Supporting Information: Figure S6B, the MTCS2 subgroup was sensitive to Imatinib, GSK269962A, A.77041, AZD.0530, KIN001.135, AMG.706, WH.4.023, CHIR.99021, lapatinib, and BIBW2992. Finally, we systematically analyzed the drug sensitivity of the five metastatic signatures in the CellMiner database, which revealed an obvious correlation between gene expression and IC_50_ values (Supporting Information: Figure S6C).

### 3.10 Verification of the classification model in the external dataset

To further confirm the robustness of the cluster results, we performed verification analysis using the JAPAN–KIRC cohort and GDSC dataset. A significant difference was observed in cell lines between MTCS1 and MTCS2 subtypes. Metastatic‐related signature expression patterns were similar between cell lines and bulk sequences. Compared with MTCS2, the MTCS1 subtype displayed increased sensitivity to tested drugs, including GSK650394, GSK690693, GSK2126458, ICL11000013, IDELALISIB, and PHA‐793887 (Supporting Information: Figure S7A). The nearest template prediction algorithm was applied to reconstruct subtypes in the JAPAN–KIRC cohort and divide this cohort into two different subgroups (Supporting Information: Figure S7B). The MTCS2 subtype in JAPAN–KIRC also displayed a poor prognosis, which was consistent with the previous results (Supporting Information: Figure S7C).

### 3.11 Construction of a subtype biomarker‐based risk model

As MTCS1 and MTCS2 exhibited distinct clinical characteristics and metastatic tendencies, we used the biomarkers of these subtypes to construct and verify a novel risk model. Univariate Cox regression analysis was used to identify survival‐related biomarkers between subtypes (Supporting Information: Figure S8A). The RF‐supervised classification algorithm identified the 10 most relevant genes (Supporting Information: Figure S8B). To establish an accurate and concise risk assessment model, we performed Kaplan–Meier analysis and calculated the p‐value for every model. We finally developed a risk assessment model composed of four signatures (ZIC2, OR13A1, CHAT, and PITX1) named RCC‐MT4 (Supporting Information: Figure S8C). The risk score of each patient was calculated as follows: RCC‐MT4  =  4.951556  × OR13A1 + 5.549985 × CHAT + 4.929467 × PITX1 + 7.868946 × ZIC2. To further identify the accuracy of RCC‐MT4, both TCGA–ccRCC and JAPAN–KIRC cohorts were divided into high‐risk and low‐risk groups according to median scores (Supporting Information: Figure S8D,E). The high‐risk group exhibited poorer OS and PFS than the low‐risk group (Supporting Information: Figure S8F). The AUC values also confirmed the high sensitivity and specificity of the RCC‐MT4 model for predicting survival outcomes. The AUC scores for the TCGA ccRCC cohort were 0.7214, 0.7546, 0.7339, 0.7193, and 0.7211 at 0.5, 1, 2, 3, and 5 years, respectively (Supporting Information: Figure S8G). Higher AUCs were obtained in the JAPAN–KIRC cohort, with values of 0.9691, 0.6351, 0.7499, 0.715, and 0.7919 at 0.5, 1, 2, 3, and 5 years, respectively (Supporting Information: Figure S8H). The above results confirmed the reliability and practicality of our classification.

### 3.12 The core role of MXRA5 in ccRCC

Given the importance of the five metastatic signatures in ccRCC, we applied multiple datasets and experiments to identify promising therapeutic targets. Combined with a single‐cell dataset from a previous study, which was composed of different stages of ccRCC tissues, we found that MXRA5 was distinctively expressed in tumor cell clusters, and its expression increased according to the progression of clinical stages (Supporting Information: Figure S9A‐C). Together, these results prompted us to determine its role in ccRCC. The immunohistochemical score of MXRA5 was increased in advanced‐stage tissues compared with that in early‐stage ccRCC tissues (*p* < 0.05) (Supporting Information: Figure S9D). The analysis of Clinical Proteomic Tumor Analysis Consortium (CPTAC) datasets further validated these results (Supporting Information: Figure S9E). Accordingly, the high expression of MXRA5 predicted the poor prognosis of ccRCC and was treated as a risk factor (Supporting Information: Figure S10). Besides, MXRA5 was highly expressed in metastatic tissues at the protein level, and the AUC reached 0.87 when evaluating the efficacy of MXRA5 in predicting ccRCC metastasis probability in the Changhai cohort (Supporting Information: Figure S9F,G). To verify the function of MXRA5 in RC cell lines, we constructed an MXRA5 knockdown lentivirus, and cell lines were transfected with negative control and MXRA5 lentivirus. Using CCK‐8 assays, knockdown of MXRA5 significantly inhibited the proliferation of ccRCC cell lines (*p* < 0.001) (Supporting Information: Figure S9H). Wound healing and clone‐forming experiments also validated that MXRA5 knockdown decreased migration and proliferation, respectively (Supporting Information: Figure S9I,J). Transwell assays showed that downregulated MXRA5 expression significantly reduced the migration ability (*p* < 0.001) (Supporting Information: Figure S9K). In vitro experiments showed that the downregulation of MXRA5 inhibited the volume and weight of tumors in mice (*p* < 0.01) (Supporting Information: Figure S9L). These results suggested that MXRA5 induced a protumorigenic phenotype in ccRCC.

## 4 DISCUSSION

Recent decades have witnessed advances in the treatment for metastatic ccRCC patients, but the 5‐year survival of these patients remains unsatisfactory [46]. Most ccRCC research projects, including TCGA, International Cancer Genome Consortium, and CPTAC, only focused on somatic mutations or cytogenetic and epigenetic alterations in primary tumors, and there are few comprehensive studies regarding the distant metastatic sites [11]. In addition, nearly 30% of ccRCC patients, even those with early clinical or pathologic stages, present with local or distant metastasis at the first diagnosis, and the prognosis of these patients is extremely poor [47]. The limitations of clinical traits (including AJCC stage, Furman score, and pathological grade) and emerging molecular alterations (e.g., VHL, PBRM1 mutation, microsatellite stage, and tumor mutation burden) hinder the capacity to provide optimal clinical management to ccRCC patients [48]. Facilitated by bioinformatics and sequencing technology, several promising prognostic models have been developed for the precision treatment of ccRCC patients. Our team proposed an RCClnc4 classifier to predict the OS of ccRCC patients in the early stages [49]. Wei et al. established six SNP‐based classifiers for predicting recurrence in localized RCC [50]. Unfortunately, these risk models fail to predict patients’ metastatic events. Thus, the identification of metastatic‐related signatures to evaluate the probability of tumor recurrence or metastasis is urgently needed.

In this study, a total of 530 metastatic DEGs between primary and metastatic sites across multiple cohorts were identified, offering a resource for the development of diagnostic and therapeutic biomarkers. Three advanced machine‐learning algorithms were applied to select the most robust metastatic signatures. Finally, five metastatic signatures (REN, ITGBL1, MXRA5, MEOX2, and ANO3) were tested in three external datasets and one out‐house dataset and displayed high accuracy in each cohort. To determine the underlying mechanism of metastasis in ccRCC, we used an unsupervised clustering algorithm to stratify ccRCC and decipher the heterogeneity of MTCS1 and MTCS2 at a multiomics level. Among the five metastatic signatures, REN is a protein‐coding gene. Diseases associated with REN include tubulointerstitial kidney disease, autosomal dominant kidney disease, and renal tubular dysgenesis. Only a few studies have revealed the role of REN in cancer. Mehranfard et al. [51] determined that REN was downregulated in colorectal cancer, which resulted in the increased production of angiotensinogen precursors of angiotensin peptides and led to the accumulation of angiotensinogen. Xie et al. [52] demonstrated an important role of local angiotensin II in the formation of an immunosuppressive TME, and its blockage might enhance tumor sensitivity to checkpoint immunotherapy. We found that REN was overexpressed in ccRCC and correlated with distant metastasis, which deserves further systematical research. ITGBL1 has been found to be overexpressed in metastatic bone cancer and reported to mediate bone metastasis in breast cancer [53]. Li et al. [54] proposed ITGBL1 as a novel metastasis‐related gene in prostate cancer, and Matsuyama et al. [55] identified ITGBL1 as a promising EMT‐associated biomarker for recurrence prediction in colorectal cancer patients, which might contribute to improved risk stratification. Cortez et al. [56] also indicated that ITGBL1 overexpression affected cellular adhesion, migration, and invasiveness, and ITGBL1‐overexpressing cells were significantly more resistant to cisplatin and paclitaxel in ovarian cancer. Huang et al. [57] determined that ITGBL1 promoted migration and invasion in hepatocellular carcinoma cells by stimulating TGF‐β/Smad signaling and functioned as a promising biomarker in hepatocellular carcinoma. Ji et al. [58] found that primary colorectal cancer released ITGBL1‐rich extracellular vesicles to promote distant metastatic tumor growth by activating the cancer‐associated fibroblast–TNFAIP–nuclear factor‐κB signaling axis. Cheli et al. [59] identified ITGBL1 as a new immunomodulator that favors the development of melanoma tumors by inhibiting natural killer cell cytotoxicity. However, little is known regarding the prognostic role of ITGBL1 in metastatic ccRCC [59]. Integrating the results from this study, we hypothesized that ITGBL1 might act as a potential indicator and therapeutic target in ccRCC management. MXRA5, a secreted glycoprotein, is a member of the MXRA protein family that participates in cell adhesion and extracellular matrix remodeling. MXRA is upregulated in human chronic ischemic myocardium and chronic kidney disease. In addition to extracellular matrix remodeling, MXRA5 has been found to be involved in tumorigenesis. Poveda et al. [60] found that MXRA5 is a TGFb1‐ and VHL‐regulated protein and functions as an anti‐inflammatory and antifibrotic molecule. He et al. [61] demonstrated that MXRA5 was aberrantly expressed in nonsmall cell lung cancer (NSCLC) tissue at the RNA and protein level and correlated with poor clinical outcomes. Xiong et al. [62] revealed that MXRA5 was the second most frequently mutated gene in Chinese patients with NSCLC and involved in the etiology of NSCLC. Sun et al. [63] also determined that MXRA5 expression was associated with clinicopathologic features and poor prognosis and played a pivotal role in the immunosuppressive microenvironment in glioma patients. However, research on the role of MXRA5 in ccRCC remains unsatisfactory. Our study found that MXRA5 was upregulated in ccRCC and might act as a biomarker to evaluate the probability of metastasis. MEOX2 belongs to the homeobox gene family and has been established as a growth arrest‐specific homeobox via cyclin‐dependent kinase inhibitor p21 and p16 activation. In a tumoral context, the dual role of MEOX2 has been reported. Reduced MEOX2 expression has been shown to be significantly correlated with short OS in hepatocellular and larynx carcinoma, whereas in lung cancer, MEOX2 overexpression has been demonstrated to be correlated with chemoresistance [64]. Tachon et al. [65] discovered that MEOX2 was one of the transcription factors associated with OS in glioma. In addition, MEOX2 contributed to cell viability and proliferation through the AKT/extracellular‐signal‐regulated kinase pathway and was involved in regulating the phenotype and adhesion properties of cancer stem cells [66]. To date, no studies have described the prognostic value of MEOX2 in ccRCC. Our study revealed the significant role of MEOX2 in ccRCC metastasis. ANO3 belongs to the TMEM16 family of predicted membrane proteins. Although there is limited information regarding the function of ANO3, Yun et al. found that ANO3 fusion was involved in breast cancer and could be treated as a novel diagnostic and therapeutic target [67].

Additionally, we enrolled the TCGA–KIRC cohort to determine the correlation between metastatic traits and intertumoral heterogeneity. Unlike previous studies, we retrieved a total of five metastatic signatures to categorize ccRCC patients. Briefly, MTCS2 was treated as a malignant phenotype with poor clinical outcomes, metastatic tendency, high expression of REN and ANO3, activated fatty acid metabolism, and activated transcripts of HNF1A, HNFAB, and EPAS1, and reduced infiltration of immune cells. In contrast, MTCS1 exhibited high expression of ITGBL1 and MXRA5, improved clinical outcomes, activated EMT, activated FOXE1, TBX18, TFE3, and TP53, and high infiltration of immune cells. Interestingly, MTCS1 was characterized by high levels of HHLA2 and RAET1E, whereas MTCS2 was associated with high levels of CD28, TMEM173, tumor necrosis factor (TNF) family members, and interleukin‐related signatures. Previous studies have indicated that HHLA2 was more frequently expressed than PDCD1 in ccRCC, and HHLA2/PDCD1 co‐expression had an adverse impact on the prognoses of patients with ccRCC, indicating that combination immunotherapy with anti‐HHLA2 and PD‐L1 blockade may function as a new strategy for ccRCC patients. Interestingly, we found that MTCS1 displayed higher co‐expression with HHL2 and PDCD1. Combined with the clinical outcomes of MTCS1, we presumed that targeting HHLA2 may be a valuable approach to improving the survival of metastatic ccRCC patients. In addition, MTCS2 displayed enhanced immune activation as the immune infiltration degree and immune‐related score calculated using multiple algorithms were significantly higher in MTCS2. The immune exhaustion score (including LAG3 and TIDE scores) was also higher in MTCS2, which indicated that a different metastatic phenotype might reverse tumor immunity and induce immune exhaustion.

Overall, MTCS2 was characterized by increased genomic instability and predominant mutations in TTN and BAP1, whereas MTCS1 displayed a lower mutation degree and mutations in PBRM1, VHL, and STD2. These results were consistent with previous reports that a high mutational load produced more neoantigens, which further induced the proliferation and activation of immune cells. In addition, the five metastatic signatures displayed no difference in mutations between MTCS1 and MTCS2, which indicated that the aberrant expression of these signatures might be regulated at the posttranscriptional level. Moreover, we found some promising targets for the high‐risk subgroup (or MTCS2). For example, the ROCK inhibitor GSK269962A was found to effectively inhibit the progression of melanoma cell lines [68], and the combination of an epidermal growth factor receptor inhibitor and GSK269926A impaired autophagosome clearance, providing a new strategy for triple‐negative breast cancer treatment [69]. A.770041, a drug inhibiting the activation of lymphocyte cell‐specific protein‐tyrosine kinase, has been demonstrated to block the invasion of highly aggressive oral cancer cells [70]. A.770041 reverses ABCB1/Pgp‐mediated chemotherapy drug resistance in osteosarcoma, and a combination of A.770041 with regular chemotherapy drugs may be clinically effective in multi‐drug resistant osteosarcoma [71]. AZD.0530 is an Src inhibitor that attenuates lactate dehydrogenase A activity through phosphorylation, thus inhibiting cancer invasion and metastasis [72]. In addition, AZD.0530 attenuated Src kinase activity and augmented PARP inhibitor‐mediated synthetic lethality in BRCA2‐altered prostate tumors [73]. Combined with the metastatic tendency of MTCS2 and drug sensitivity differences, we hypothesized that these molecular drugs may provide new therapeutic agents for metastatic ccRCC patients. The conventional ccRCC staging system failed to involve metastatic signature, which might lead to underlying insufficient treatment. Combined the heterogeneity and distinctive clinical characteristics in MTCS1 and MTCS2, we constructed and verified a novel metastatic related risk model in TCGA‐KIRC and JAPAN‐KIRC cohorts. Considering the heterogeneities (e.g., sequencing platform, technician, tissue specimen and RNA degradation) among datasets, we utilized the median value as the cutoff of risk model, which divided patients into high‐ and low‐risk groups. Interestingly, such a cut‐off value reached satisfactory performance in both training and test cohorts, which could receive a higher accuracy when considering such heterogeneous factors mentioned above and utilizing an optimal cut off value. Further prospective multi‐center cohorts should be enrolled to further verify its reproductivity and sensitivity.

To the best of our knowledge, this is the first study to identify ccRCC metastatic biomarkers and decipher metastatic tendency using multiple cohorts and multi‐omics analysis. Although the implication of the five metastatic signatures has been identified and verified, there are still some limitations. First, the detection array of different microarray platforms might differ, potentially leading to overlooking some signatures related to metastasis. Second, some molecular and clinical characteristics of public datasets were inadequate, which might have concealed the potential associations between ccRCC subtypes and certain variables. Third, the detailed role of MXRA5 in ccRCC and the TME remains unknown, and further in vivo and in vitro experiments are needed to reveal its function.

## 5 CONCLUSION

In summary, the present study identified the most pivotal signatures involved in ccRCC metastasis and identified two distinct metastatic subtypes using a multiomics approach. Our study might shed light on metastatic ccRCC and provide a significant clinical reference for risk stratification, therapeutic strategies, and prognosis prediction among ccRCC patients.

## AUTHOR CONTRIBUTIONS

**Aimin Jiang:** Conceptualization; methodology; formal analysis; and visualization. **Qingyang Pang:** Formal analysis. **Xinxin Gan:** Conceptualization. **Anbang Wang:** Methodology. **Zhenjie Wu:** Methodology. **Bing Liu:** Methodology. **Peng Luo:** Conceptualization. **Le Qu:** Supervision. **Linhui Wang:** Resources; supervision; and writing – review and editing.

## CONFLICT OF INTEREST

The authors declare no conflict of interest.

## ETHICS STATEMENT

The procedure related to human subjects was approved by the Ethics Committee of the Changhai Hospital (CHEC2022074). All animal experiments were approved by the Animal Ethics Committee of the Changhai Hospital (CHEC2021‐191).

## INFORMED CONSENT

Informed consent was obtained from all subjects to use the specimens described in this study.

## Supporting information

Supporting information.

Supporting information.

## Data Availability

The patient data in this study were acquired from publicly available datasets with complete informed patient consent.
