# Supplementary material for: Definition and verification of novel metastasis and recurrence related signatures of ccRCC: A multicohort study
Source: Cancer Innov. 2022 Aug 30;1(2):146–67. doi: 10.1002/cai2.25 (PMC10686128; doi:10.1002/cai2.25)
Supplement: Supplementary file 1 — Supporting information. [file CAI2-1-146-s001.pdf]

Figure S1

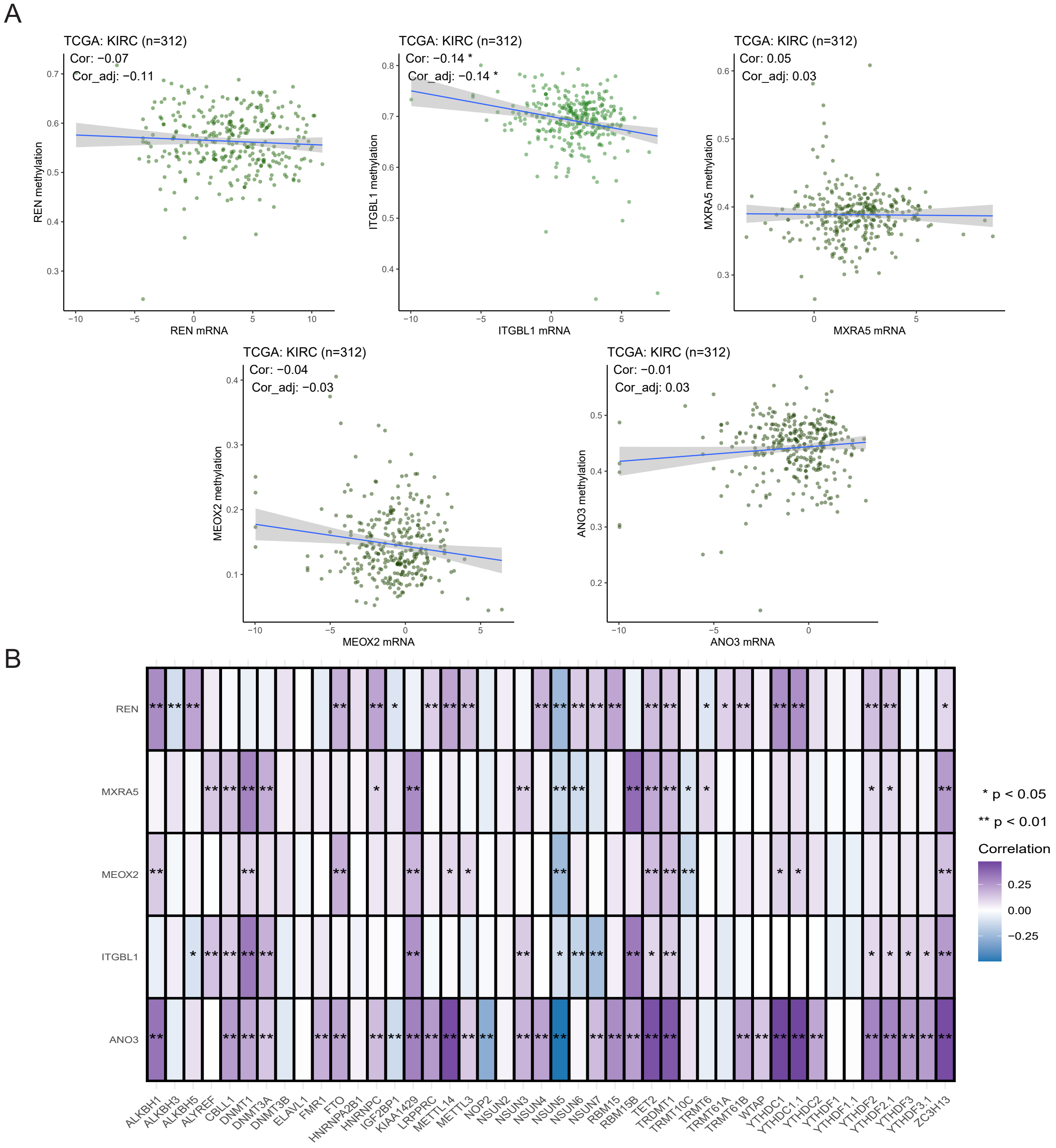
A
TCGA: KIRC (n=312)
Cor: −0.07
Cor_adj: −0.11
REN methylation
REN mRNA
TCGA: KIRC (n=312)
Cor: −0.14 *
Cor_adj: −0.14 *
ITGBL1 methylation
ITGBL1 mRNA
TCGA: KIRC (n=312)
Cor: 0.05
Cor_adj: 0.03
MXRA5 methylation
MXRA5 mRNA
TCGA: KIRC (n=312)
Cor: −0.04
Cor_adj: −0.03
MEOX2 methylation
MEOX2 mRNA
TCGA: KIRC (n=312)
Cor: −0.01
Cor_adj: 0.03
ANO3 methylation
ANO3 mRNA
B
REN
MXRA5
MEOX2
ITGBL1
ANO3
ALKBH1 ALKBH3 ALKBH5 ALYREF CBLL1 DNMT1 DNMT3A DNMT3B ELAVL1 FMR1 FTO HNRNPA2B1 HNRNPC IGF2BP1 KIAA1429 LRPPRC METTL14 METTL3 NOP2 NSUN2 NSUN3 NSUN4 NSUN5 NSUN6 NSUN7 RBM15 RBM15B TET2 TRDMT1 TRMT10C TRMT6 TRMT61A TRMT61B WTAP YTHDC1 YTHDC1.1 YTHDC2 YTHDF1 YTHDF1.1 YTHDF2 YTHDF2.1 YTHDF3 YTHDF3.1 ZC3H13
* p < 0.05
** p < 0.01
Correlation
0.25
0.00
−0.25

Figure S2

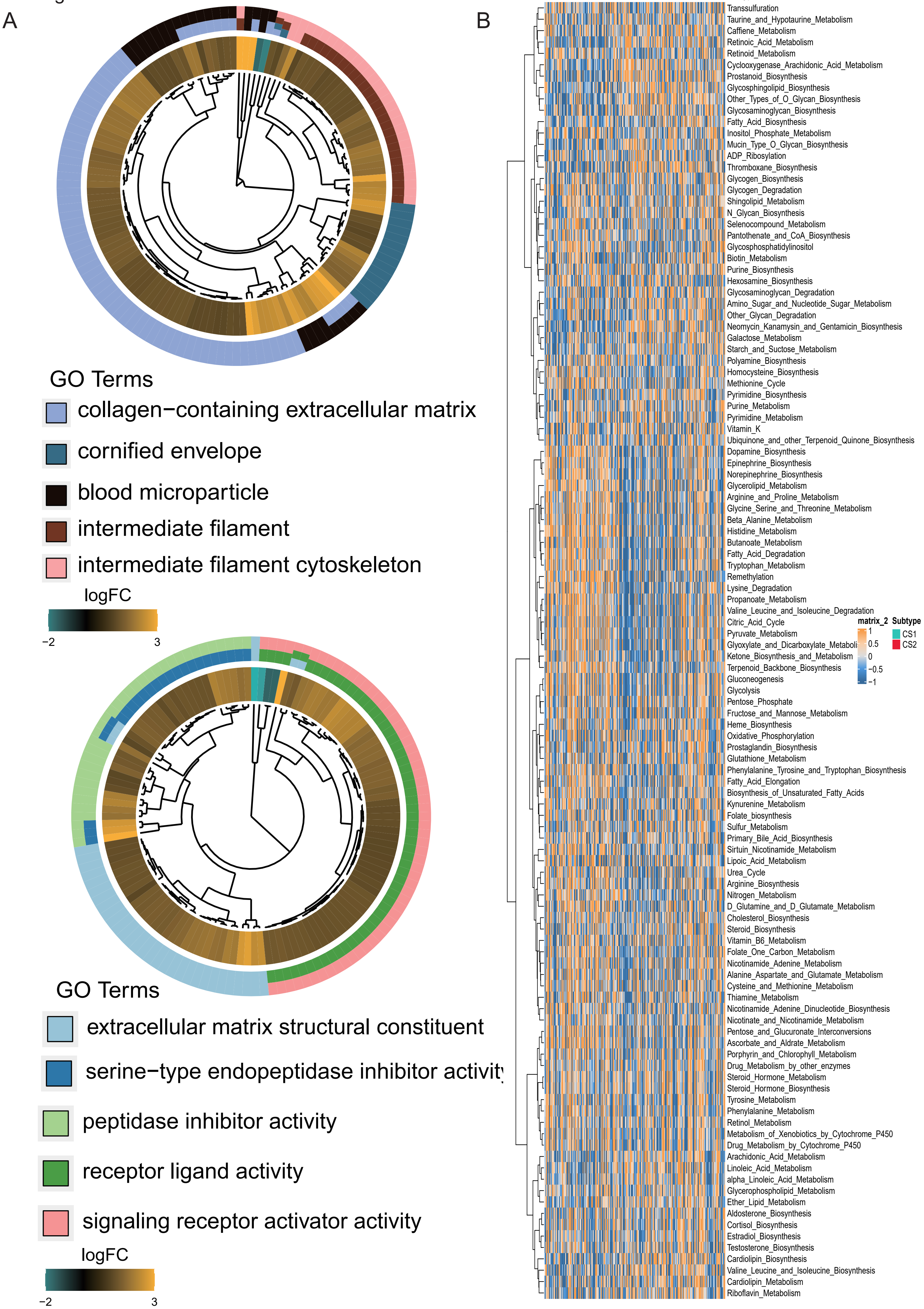

A
GO Terms
collagen−containing extracellular matrix
cornified envelope
blood microparticle
intermediate filament
intermediate filament cytoskeleton
logFC
−2
3
GO Terms
extracellular matrix structural constituent
serine−type endopeptidase inhibitor activity
peptidase inhibitor activity
receptor ligand activity
signaling receptor activator activity
logFC
−2
3
B
Subtype
Transsulfuration
Taurine_and_Hypotaurine_Metabolism
Caffiene_Metabolism
Retinoic_Acid_Metabolism
Retinoid_Metabolism
Cyclooxygenase_Arachidonic_Acid_Metabolism
Prostanoid_Biosynthesis
Glycosphingolipid_Biosynthesis
Other_Types_of_O_Glycan_Biosynthesis
Glycosaminoglycan_Biosynthesis
Fatty_Acid_Biosynthesis
Inositol_Phosphate_Metabolism
Mucin_Type_O_Glycan_Biosynthesis
ADP_Ribosylation
Thromboxane_Biosynthesis
Glycogen_Biosynthesis
Glycogen_Degradation
Shingolipid_Metabolism
N_Glycan_Biosynthesis
Selenocompound_Metabolism
Pantothenate_and_CoA_Biosynthesis
Glycosphosphatidylinositol
Biotin_Metabolism
Purine_Biosynthesis
Hexosamine_Biosynthesis
Glycosaminoglycan_Degradation
Amino_Sugar_and_Nucleotide_Sugar_Metabolism
Other_Glycan_Degradation
Neomycin_Kanamysin_and_Gentamicin_Biosynthesis
Galactose_Metabolism
Starch_and_Suctose_Metabolism
Polyamine_Biosynthesis
Homocysteine_Biosynthesis
Methionine_Cycle
Pyrimidine_Biosynthesis
Purine_Metabolism
Pyrimidine_Metabolism
Vitamin_K
Ubiquinone_and_other_Terpenoid_Quinone_Biosynthesis
Dopamine_Biosynthesis
Epinephrine_Biosynthesis
Norepinephrine_Biosynthesis
Glycerolipid_Metabolism
Arginine_and_Proline_Metabolism
Glycine_Serine_and_Threonine_Metabolism
Beta_Alanine_Metabolism
Histidine_Metabolism
Butanoate_Metabolism
Fatty_Acid_Degradation
Tryptophan_Metabolism
Remethylation
Lysine_Degradation
Propanoate_Metabolism
Valine_Leucine_and_Isoleucine_Degradation
Citric_Acid_Cycle
Pyruvate_Metabolism
Glyoxylate_and_Dicarboxylate_Metaboli
Ketone_Biosynthesis_and_Metabolism
Terpenoid_Backbone_Biosynthesis
Gluconeogenesis
Glycolysis
Pentose_Phosphate
Fructose_and_Mannose_Metabolism
Heme_Biosynthesis
Oxidative_Phosphorylation
Prostaglandin_Biosynthesis
Glutathione_Metabolism
Phenylalanine_Tyrosine_and_Tryptophan_Biosynthesis
Fatty_Acid_Elongation
Biosynthesis_of_Unsaturated_Fatty_Acids
Kynurenine_Metabolism
Folate_biosynthesis
Sulfur_Metabolism
Primary_Bile_Acid_Biosynthesis
Sirtuin_Nicotinamide_Metabolism
Lipoic_Acid_Metabolism
Urea_Cycle
Arginine_Biosynthesis
Nitrogen_Metabolism
D_Glutamine_and_D_Glutamate_Metabolism
Cholesterol_Biosynthesis
Steroid_Biosynthesis
Vitamin_B6_Metabolism
Folate_One_Carbon_Metabolism
Nicotinamide_Adenine_Metabolism
Alanine_Aspartate_and_Glutamate_Metabolism
Cysteine_and_Methionine_Metabolism
Thiamine_Metabolism
Nicotinamide_Adenine_Dinucleotide_Biosynthesis
Nicotinate_and_Nicotinamide_Metabolism
Pentose_and_Glucuronate_Interconversions
Ascorbate_and_Aldrate_Metabolism
Porphyrin_and_Chlorophyll_Metabolism
Drug_Metabolism_by_other_enzymes
Steroid_Hormone_Metabolism
Steroid_Hormone_Biosynthesis
Tyrosine_Metabolism
Phenylalanine_Metabolism
Retinol_Metabolism
Metabolism_of_Xenobiotics_by_Cytochrome_P450
Drug_Metabolism_by_Cytochrome_P450
Arachidonic_Acid_Metabolism
Linoleic_Acid_Metabolism
alpha_Linoleic_Acid_Metabolism
Glycerophospholipid_Metabolism
Ether_Lipid_Metabolism
Aldosterone_Biosynthesis
Cortisol_Biosynthesis
Estradiol_Biosynthesis
Testosterone_Biosynthesis
Cardiolipin_Biosynthesis
Valine_Leucine_and_Isoleucine_Biosynthesis
Cardiolipin_Metabolism
Riboflavin_Metabolism
matrix_2
1
0.5
0
−0.5
−1
Subtype
CS1
CS2

Figure S3

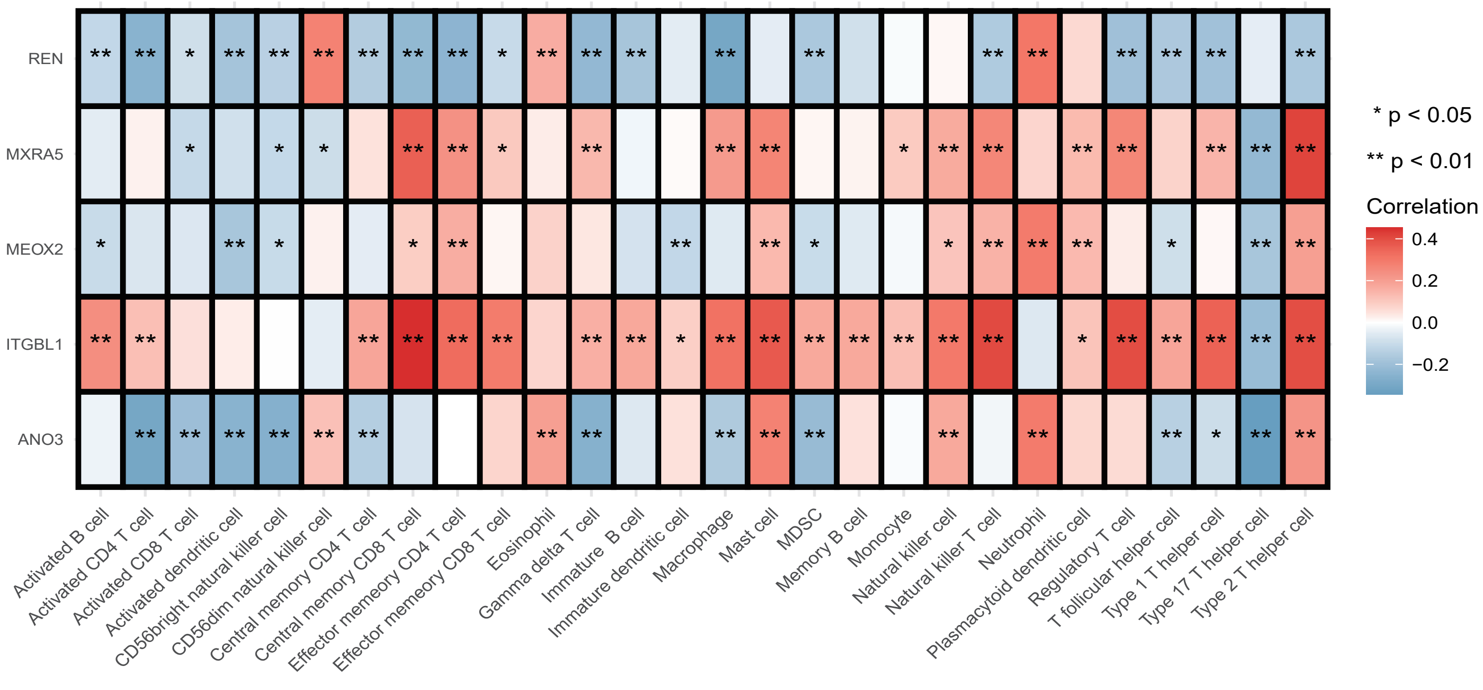

REN
MXRA5
MEOX2
ITGBL1
ANO3
Activated B cell
Activated CD4 T cell
Activated CD8 T cell
Activated dendritic cell
CD56bright natural killer cell
CD56dim natural killer cell
Central memory CD4 T cell
Central memory CD8 T cell
Effector memeory CD4 T cell
Effector memeory CD8 T cell
Eosinophil
Gamma delta T cell
Immature B cell
Immature dendritic cell
Macrophage
Mast cell
MDSC
Memory B cell
Monocyte
Natural killer cell
Natural killer T cell
Neutrophil
Plasmacytoid dendritic cell
Regulatory T cell
T follicular helper cell
Type 1 T helper cell
Type 17 T helper cell
Type 2 T helper cell
* p < 0.05
** p < 0.01
Correlation
0.4
0.2
0.0
−0.2

Figure S4

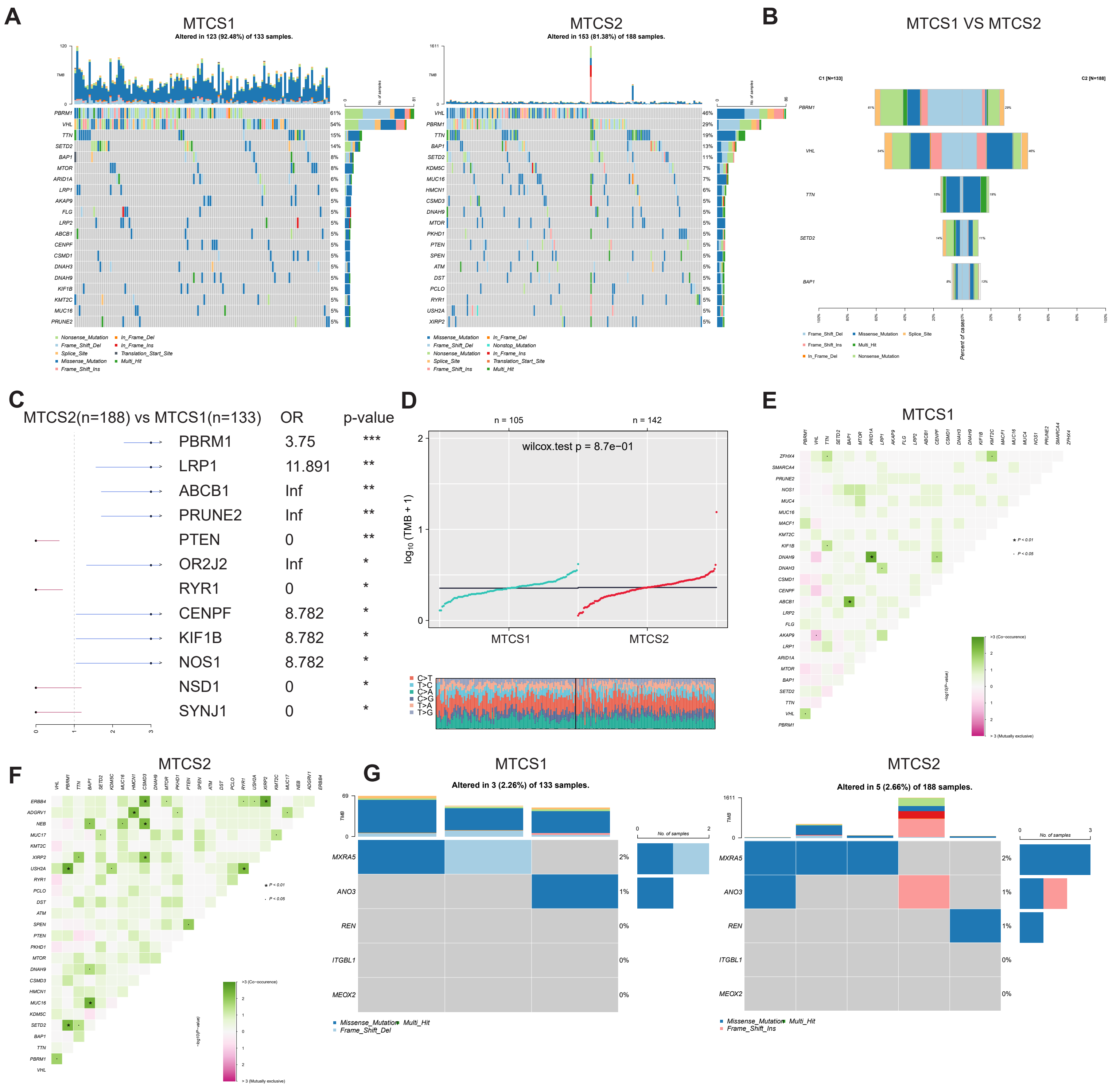

A
MTCS1
Altered in 123 (92.48%) of 133 samples.
MTCS2
Altered in 153 (81.38%) of 188 samples.
B
MTCS1 VS MTCS2
C
MTCS2(n=188) vs MTCS1(n=133)
OR
p-value
PBRM1 3.75 ***
LRP1 11.891 **
ABCB1 Inf **
PRUNE2 Inf **
PTEN 0 **
OR2J2 Inf *
RYR1 0 *
CENPF 8.782 *
KIF1B 8.782 *
NOS1 8.782 *
NSD1 0 *
SYNJ1 0 *
D
n = 105
n = 142
wilcox.test p = 8.7e−01
MTCS1
MTCS2
E
MTCS1
F
MTCS2
G
MTCS1
Altered in 3 (2.26%) of 133 samples.
MTCS2
Altered in 5 (2.66%) of 188 samples.

Figure S5

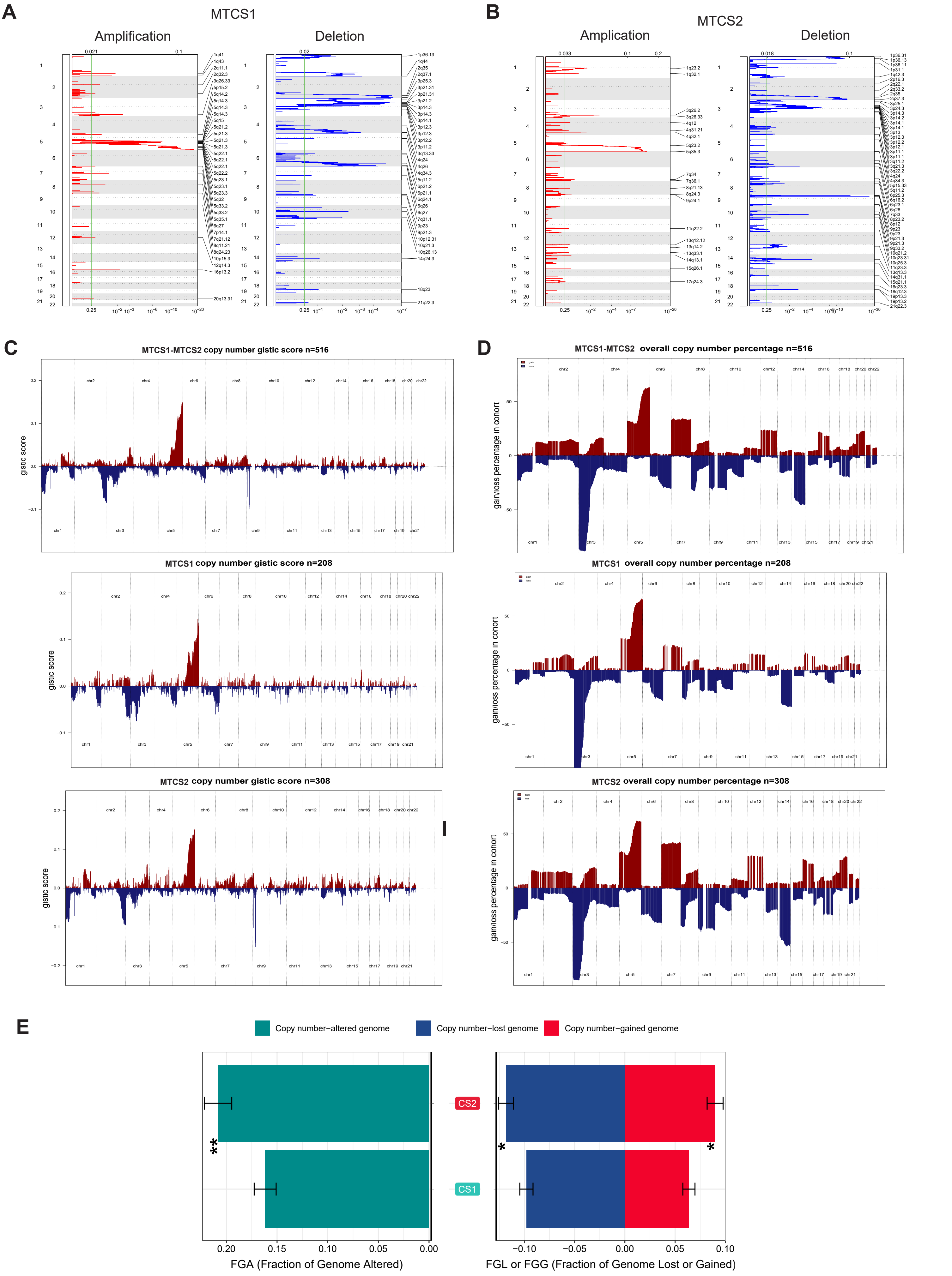

A
MTCS1
Amplification
Deletion
B
MTCS2
Amplification
Deletion
C
MTCS1-MTCS2 copy number gistic score n=516
MTCS1 copy number gistic score n=208
MTCS2 copy number gistic score n=308
gistic score
D
MTCS1-MTCS2 overall copy number percentage n=516
MTCS1 overall copy number percentage n=208
MTCS2 overall copy number percentage n=308
gain/loss percentage in cohort
E
Copy number−altered genome
Copy number−lost genome
Copy number−gained genome
CS2
CS1
FGA (Fraction of Genome Altered)
FGL or FGG (Fraction of Genome Lost or Gained)

Figure S6

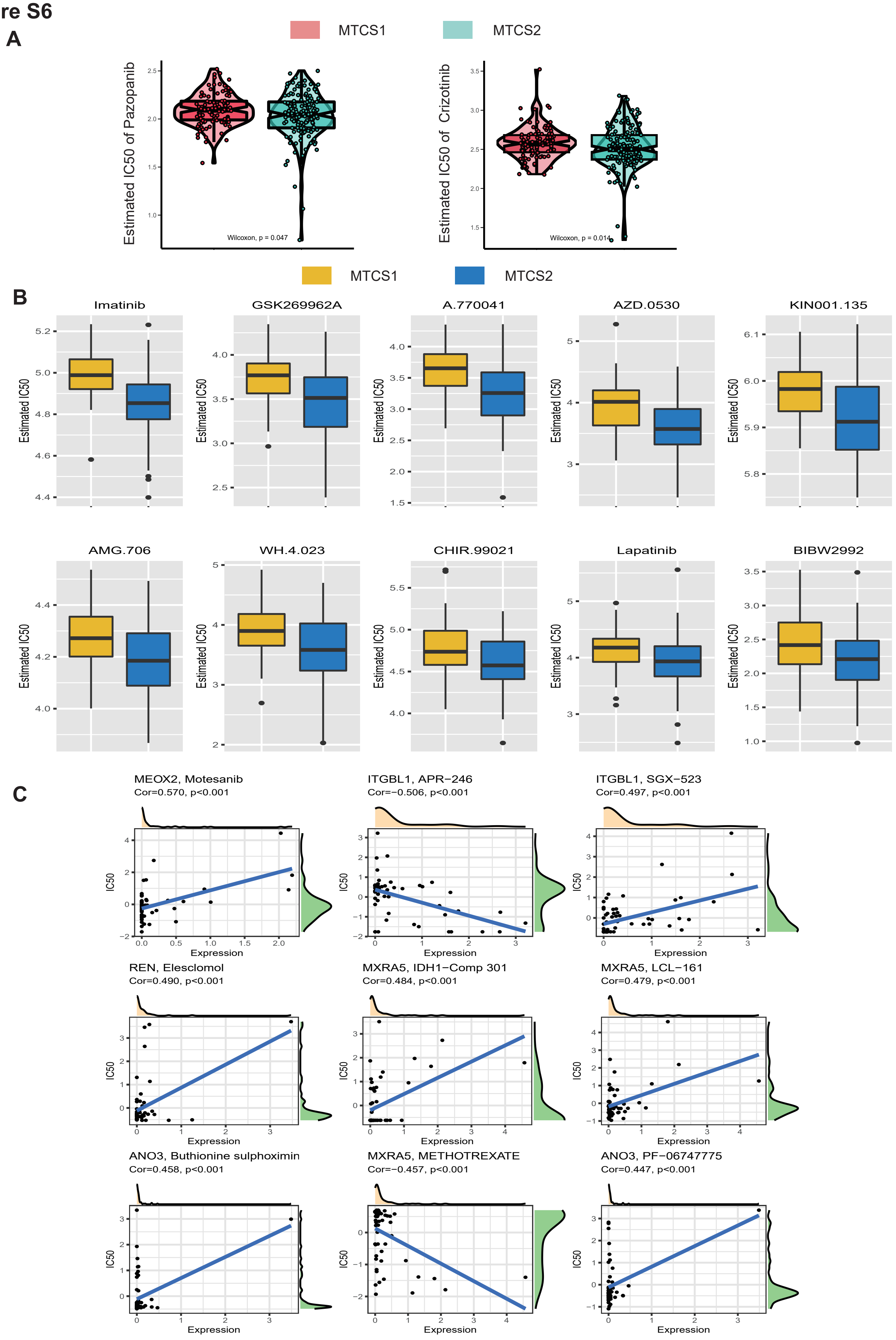
A
MTCS1
MTCS2
Estimated IC50 of Pazopanib
Wilcoxon, p = 0.047
Estimated IC50 of Crizotinib
Wilcoxon, p = 0.014
B
MTCS1
MTCS2
Imatinib
GSK269962A
A.770041
AZD.0530
KIN001.135
AMG.706
WH.4.023
CHIR.99021
Lapatinib
BIBW2992
Estimated IC50
C
MEOX2, Motesanib
Cor=0.570, p<0.001
ITGBL1, APR−246
Cor=−0.506, p<0.001
ITGBL1, SGX−523
Cor=0.497, p<0.001
REN, Elesclomol
Cor=0.490, p<0.001
MXRA5, IDH1−Comp 301
Cor=0.484, p<0.001
MXRA5, LCL−161
Cor=0.479, p<0.001
ANO3, Buthionine sulphoximin
Cor=0.458, p<0.001
MXRA5, METHOTREXATE
Cor=−0.457, p<0.001
ANO3, PF−06747775
Cor=0.447, p<0.001
IC50
Expression

Figure S7

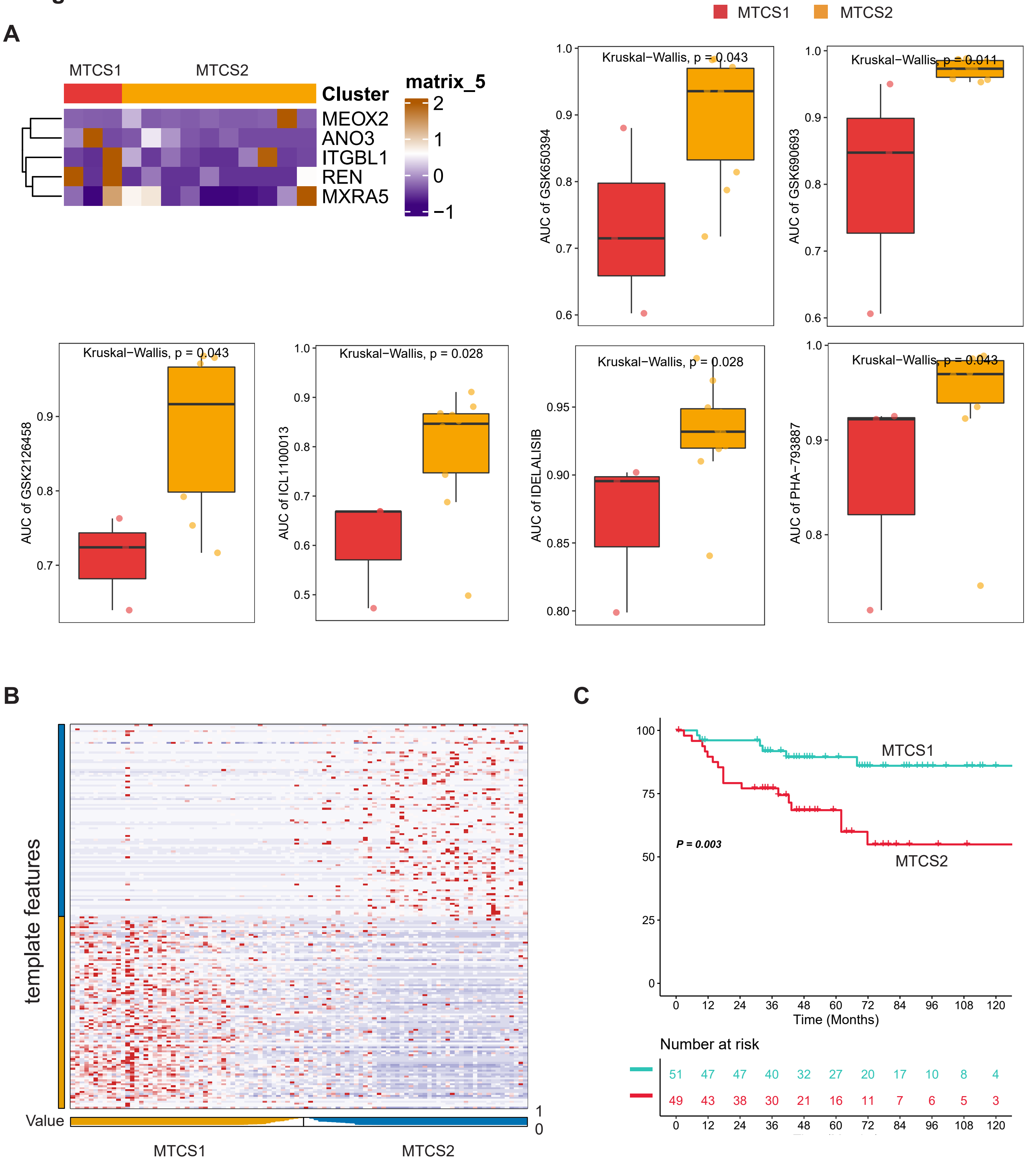
A
MTCS1
MTCS2
Cluster
matrix_5
2
1
0
−1
MEOX2
ANO3
ITGBL1
REN
MXRA5
Kruskal−Wallis, p = 0.043
AUC of GSK650394
Kruskal−Wallis, p = 0.011
AUC of GSK690693
Kruskal−Wallis, p = 0.043
AUC of GSK2126458
Kruskal−Wallis, p = 0.028
AUC of ICL1100013
Kruskal−Wallis, p = 0.028
AUC of IDELALISIB
Kruskal−Wallis, p = 0.043
AUC of PHA−793887
B
template features
Value
MTCS1
MTCS2
C
MTCS1
MTCS2
P = 0.003
Time (Months)
Number at risk
51 47 47 40 32 27 20 17 10 8 4
49 43 38 30 21 16 11 7 6 5 3
0 12 24 36 48 60 72 84 96 108 120

Figure S8

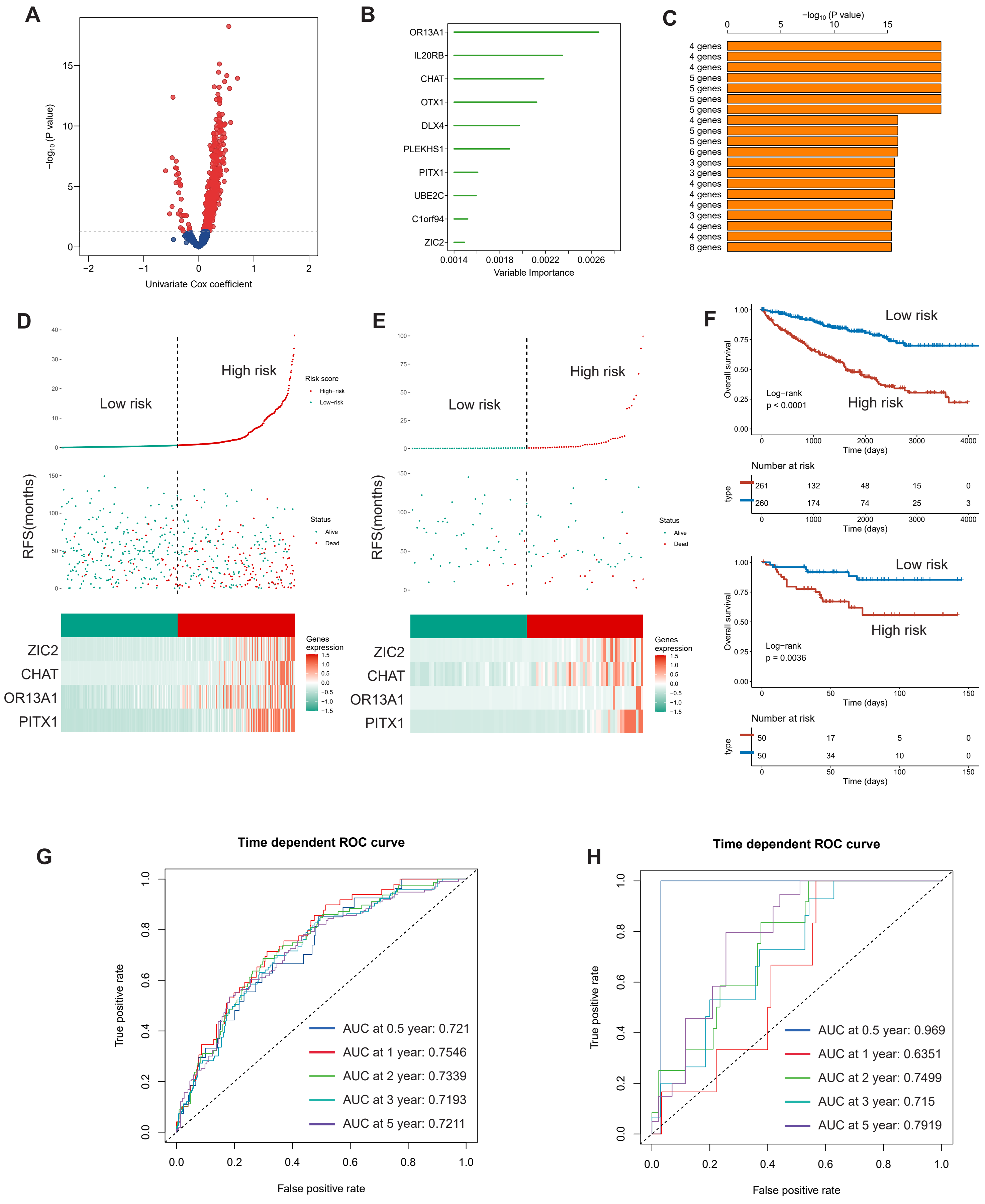
A
B
C
D
E
F
G
H
Univariate Cox coefficient
−log10 (P value)
Variable Importance
OR13A1
IL20RB
CHAT
OTX1
DLX4
PLEKHS1
PITX1
UBE2C
C1orf94
ZIC2
Low risk
High risk
Risk score
High−risk
Low−risk
RFS(months)
Status
Alive
Dead
Genes expression
Overall survival
Log−rank
p < 0.0001
p = 0.0036
Time (days)
Number at risk
type
Time dependent ROC curve
True positive rate
False positive rate
AUC at 0.5 year: 0.721
AUC at 1 year: 0.7546
AUC at 2 year: 0.7339
AUC at 3 year: 0.7193
AUC at 5 year: 0.7211
AUC at 0.5 year: 0.969
AUC at 1 year: 0.6351
AUC at 2 year: 0.7499
AUC at 3 year: 0.715
AUC at 5 year: 0.7919

Figure S9

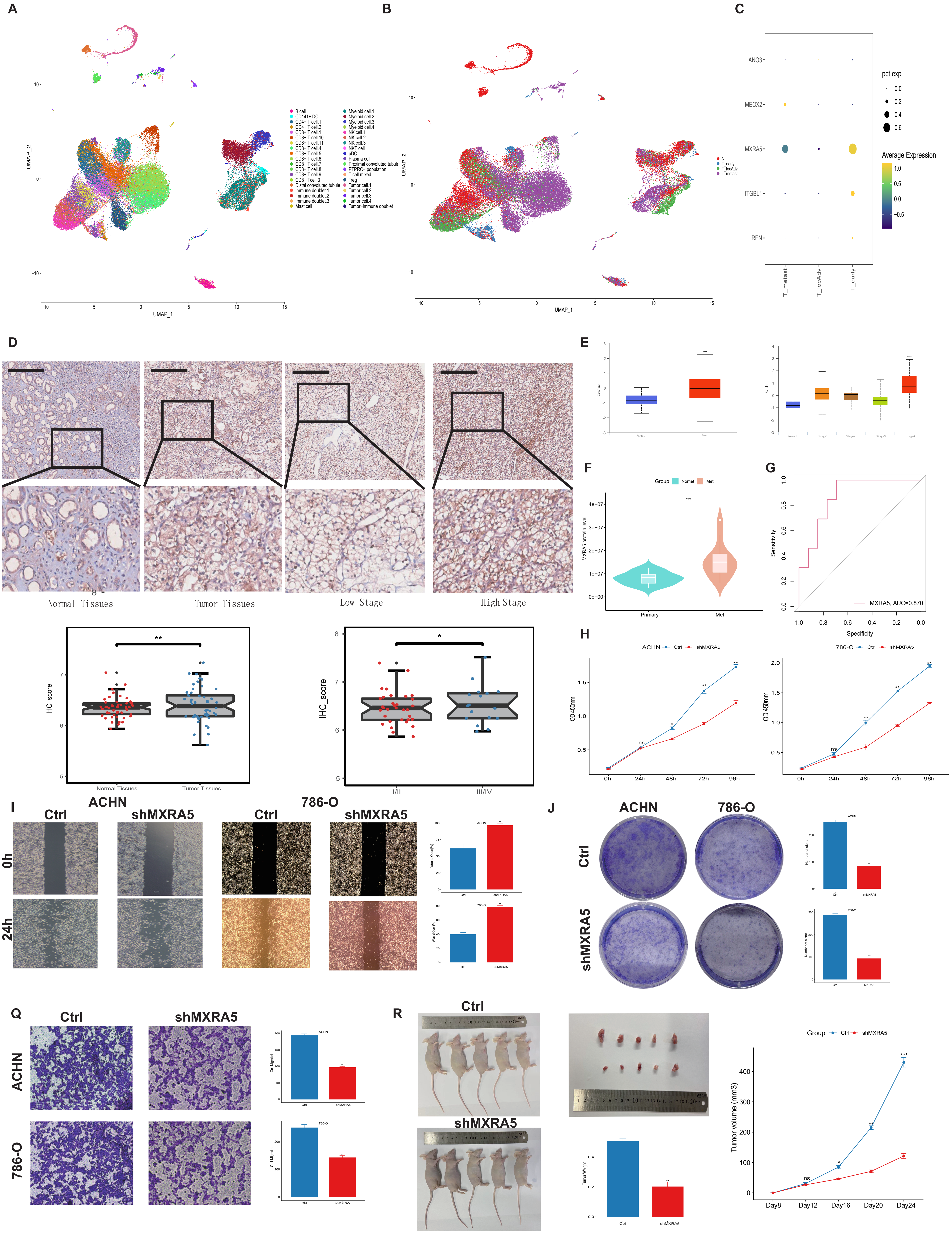
A
B
C
D
E
F
G
H
I
J
Q
R
UMAP_1
UMAP_2
pct.exp
Average Expression
ANO3
MEOX2
MXRA5
ITGBL1
REN
T_metast
T_locAdv
T_early
Normal Tissues
Tumor Tissues
Low Stage
High Stage
IHC_score
I/II
III/IV
Group
Nomet
Met
MXRA5 protein level
Primary
Sensitivity
Specificity
MXRA5, AUC=0.870
ACHN
786-O
Ctrl
shMXRA5
OD 450nm
0h
24h
48h
72h
96h
Tumor volume (mm3)
Tumor Weight
Day8
Day12
Day16
Day20
Day24

Figure S9

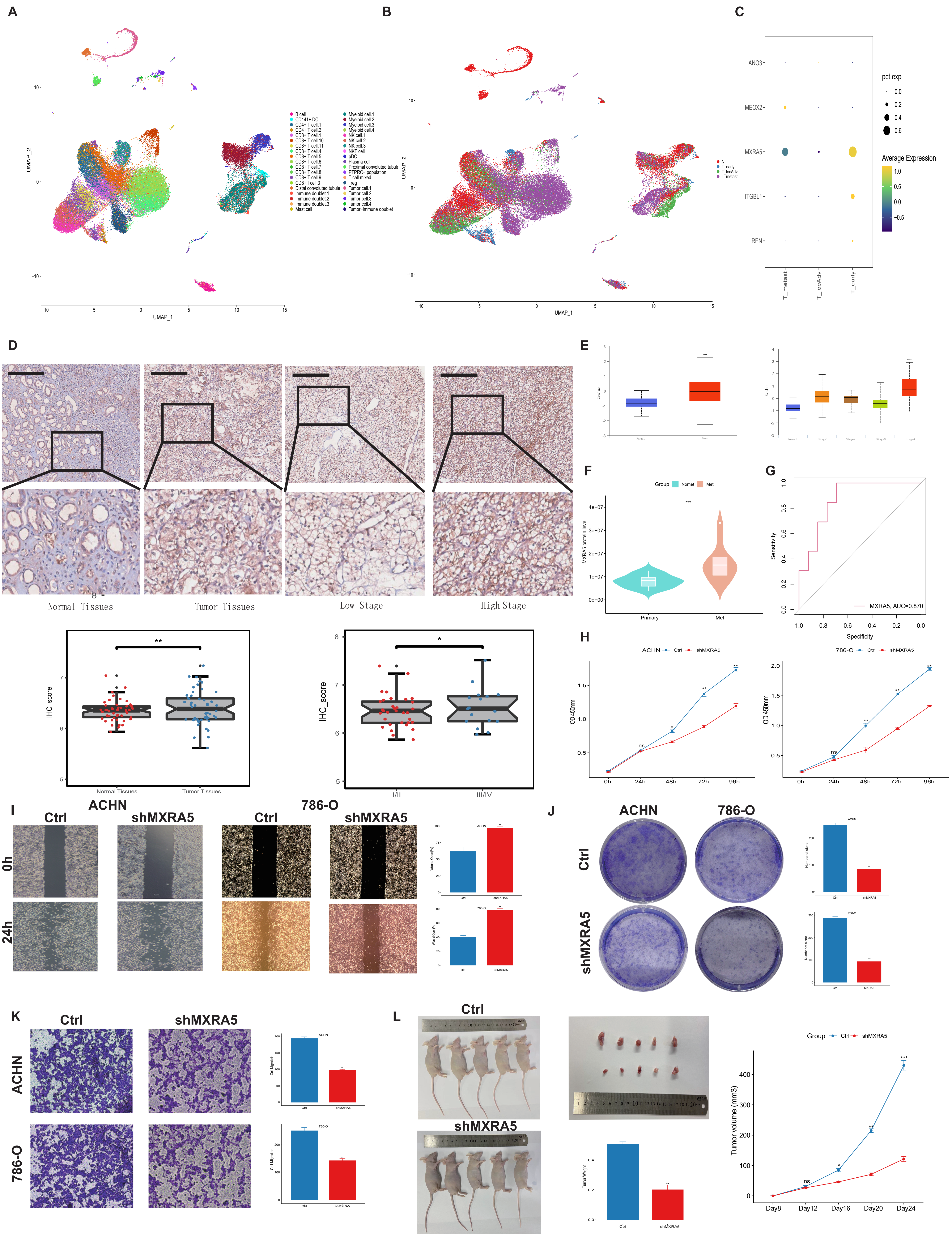

A
B
C
D
E
F
G
H
I
J
K
L
UMAP_1
UMAP_2
Normal Tissues
Tumor Tissues
Low Stage
High Stage
IHC_score
ACHN
786-O
Ctrl
shMXRA5
0h
24h
MXRA5, AUC=0.870
Sensitivity
Specificity
OD 450nm
Tumor volume (mm3)
Group

Figure S10

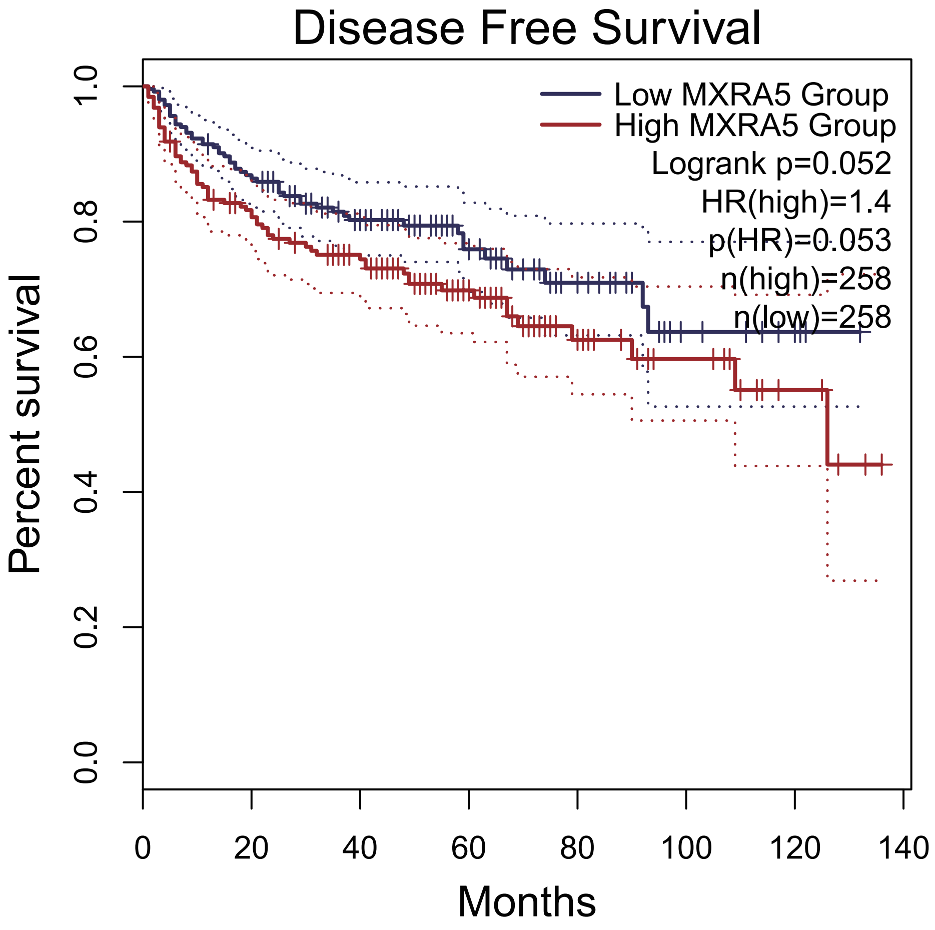

Disease Free Survival
Low MXRA5 Group
High MXRA5 Group
Logrank p=0.052
HR(high)=1.4
p(HR)=0.053
n(high)=258
n(low)=258
Percent survival
0.0
0.2
0.4
0.6
0.8
1.0
0
20
40
60
80
100
120
140
Months
