# Supplementary material for: Definition and verification of novel metastasis and recurrence related signatures of ccRCC: A multicohort study
Source: Cancer Innov. 2022 Aug 30;1(2):146–67. doi: 10.1002/cai2.25 (PMC10686128; doi:10.1002/cai2.25)
Supplement: Supplementary file 2 — Supporting information. [file CAI2-1-146-s002.docx]

**Supplementary Figures:**

**Figure S1:** **DNA methylation and RNA modification of five metastasis related signatures**

(A) The correlation of five metastasis related signatures expression and methylation degree in ccRCC. (B) The correlation of five metastasis related signatures expression and RNA modification regulator expression in ccRCC.

**Figure S2: Functional enrichment analysis of ccRCC subtypes.**

(A) GO enrichment analysis of CC and MF. (B)GSVA analysis of metabolism pathways between subtypes.

**Figure S3: Correlation of five metastasis related signatures and immune cell infiltration in ccRCC.**

**Figure S4: Landscapes of somatic mutations and potential targets in** **two subtypes**

(A) Waterfall plot showing the mutation patterns of the top 20 most frequently mutated genes in MTCS1 and MTCS2. (B) Bar plot displaying the top 6 most significantly differentially mutated genes between subgroups. (C) Forest plot indicating the prognostic difference of mutated genes between subtypes. (D) Difference of TMB between subgroups. (E, F) Heatmap illustrates the mutually cooccurring and exclusive mutations of the top 25 frequently mutated genes in MTCS1 and MTCS2. The color and symbol in each cell represent the statistical significance of the exclusivity or cooccurrence for each pair of genes. (G) Waterfall plot shows the mutation patterns of the five metastasis signatures in MTCS1 and MTCS2.

**Figure S5: Landscapes of copy number variations in two subtypes**

(A, B) The GISTIC score and percentage of copy number profiles in ccRCC. (C, D) Composite copy number profiles for ccRCC with gains in red and losses in blue and gray highlighting differences. (E) Bar-plot of genomic fractions altered between subtypes.

**Figure S6: Verification of classification model in external dataset**

(A) Heatmap of the expression profiles of five metastasis signatures in the two subtypes of renal cancer cell lines; and drug sensitivity values of each cell line subtypes. (B) Heatmap of NTP in JAPAN-KIRC cohort using subtype-specific upregulated hallmarks identified from MTCS1 and MTCS2. (C) Survival analysis of the two predicted subtypes of ccRCC in JAPAN-KIRC cohort.

**Figure S7: Drug sensitivity analysis of two subtypes**

(A) Estimated IC50 of the indicated molecular targeted drugs and (B) potential drugs between subgroups. (C) Correlation between five metastasis related signatures and sensitivity of the top 9 anticancer drugs in CellMiner database.

**Figure S8: Construction of a risk model based on subtypes’ biomarker**

(A) Volcano plot showing the biomarkers between the two subtypes by univariable Cox regression analysis. (B) Random survival forest analysis screening ten genes. (C) Basing various combination analyses, the top 20 signatures are ordered by the p-value. (D, E) Risk score analysis in TCGA-ccRCC and JAPAN-KIRC cohorts. (F) Survival analysis of the two risk signatures in TCGA-ccRCC and JAPAN-KIRC cohorts. (G, H) The time-dependent ROC curves for the two risk signatures in TCGA-ccRCC and JAPAN-KIRC cohorts.

**Figure S9: The core role of MXRA5 and functional verification**

(A, B) The t-SNE plot shows total cells divided into different clusters and clinical stage. (C) Expression level of five metastasis related signatures at single cell level. (D) Immunohistochemical score of MXRA5 in normal vs. tumor tissues and low-stage vs. high-stage ccRCC. (E) Difference expression of MXRA5 in ccRCC at protein level. (F) Difference expression of MXRA5 in ccRCC at protein level in Changhai cohort. (G)AUC curve of MXRA5 in predicant ccRCC metastasis. (H)Cell proliferation, migration (I), clone formation ability (J) and invasion (K) of ACHN and 786-O after being transfected with NC and sh-MXRA5 lentivirus. (L) Subcutaneous xenograft models were established, and tumor weight and growth curve of ACHN cells infected of NC and sh-MXRA5 lentivirus.

**Figure S10: Survive plot of ccRCC based on MXRA5 expression level.**
